# Impact of the pandemic and its containment measures in Europe upon aspects of affective impairments: a Google Trends informetrics study

**DOI:** 10.1017/S0033291723001563

**Published:** 2023-12

**Authors:** Istvan-Szilard Szilagyi, Eva Eggeling, Helmar Bornemann-Cimenti, Torsten Ullrich

**Affiliations:** 1 Department of Anesthesiology and Intensive Care Medicine, Medical University of Graz, 8036 Graz, Austria; 2 Department of Medical Psychology and Psychotherapy, Medical University of Graz, 8036 Graz, Austria; 3 Fraunhofer Austria Research GmbH, 8010 Graz, Austria; 4 Institute of Computer Graphics and Knowledge Visualization, Graz University of Technology, 8010 Graz, Austria

**Keywords:** Affective impairments, COVID-19, data science, internet medicine, pandemic, Psychology

## Abstract

**Background:**

In late 2019, a new virus began spreading in Wuhan, China. By the end of 2021, more than 260 million people worldwide had been infected and 5.2 million people had died because of the severe acute respiratory syndrome coronavirus 2 (SARS-CoV-2). Various countermeasures have been implemented to contain the infections, depending on the country, infection prevalence, and political and infrastructural resources. The pandemic and the containment measures have induced diverse psychological burdens. Using internet queries as a proxy, this study examines the psychological consequences on a European level of SARS-CoV-2 containment measures.

**Methods:**

Using informetric analyses, this study reviews within 32 European countries a total of 28 search parameters derived from the International Statistical Classification of Diseases and Related Health Problems (ICD-10) as aspects of affective disorder.

**Results:**

Our results show that there are several psychological aspects which are significantly emphasized during the pandemic and its containment measures: ‘anxiety’, ‘dejection’, ‘weariness’, ‘listlessness’, ‘loss of appetite’, ‘loss of libido’, ‘panic attack’, and ‘worthlessness’. These terms are significantly more frequently part of a search query during the pandemic than before the outbreak. Furthermore, our results revealed that search parameters such as ‘psychologist’, ‘psychotherapist’, ‘psychotherapy’ have increased highly significantly (*p* < 0.01) since the pandemic.

**Conclusions:**

The psychological distress caused by the pandemic correlates significantly with the frequency of people searching for psychological and psychotherapeutic support on the Internet.

## Introduction

In late 2019, a new infectious disease began to spread in China in the city of Wuhan, resulting in fever, severe respiratory symptoms, fatigue, gastrointestinal and neurological problems, and other serious conditions, often with a lethal outcome (Fernández-de-las-Peñas et al., 2021). This single-stranded RNA virus was named ‘corona virus’ or SARS-CoV-2 (COVID-19) by the World Health Organization (WHO) because of its spiky form. As of March 2020, the WHO declared the outbreak of SARS-CoV-2 to be a pandemic (WHO, 2021*b*). The virus has an insidious characteristic because it spreads very rapidly, undergoes rapid mutations, is transmissible easily from person to person through droplet infection and physical contact, and can affect all people, regardless of age. By March 2020, the number of infections outside China was already increasing exponentially. By the end of November 2021, more than 260 million people worldwide had already been infected and 5.2 million people had died due to consequences associated with SARS-CoV-2 (Baloch, Baloch, Zheng, & Pei, 2020; WHO, 2021*a*; Worldometers.info, 2021). Higher risk for major complications, and even ultimately for death, due to SARS-CoV-2 infection is seen in elderly patients, in patients with comorbid diseases, and among patients who have been impaired by immunodeficiency.

Various countermeasures have been introduced to contain COVID-19 infections, depending on country, infection prevalence, and political and infrastructural resources. The set of measures includes home-based work, home schooling, closure of stores and public places, travel restrictions, and even a complete lockdown of social life. These interventions resulted in a reduction of infection rates in countries where measures were taken compared to those where not. The effectiveness of the lockdown measures, in particular, continue to show a beneficial trend toward decreasing infection rates at least 20 days after the end of the measurement (Alfano & Ercolano, 2020). Although these measures were successful, their consequences in economic, environmental, and psychological terms quickly became apparent. In particular, the psychological consequences proved to be profound. Thus, the pandemic as well as the applied measures had unfavorable psychological consequences, especially in the psycho-affective sphere. Increases in generalized anxiety, isolated phobias, depression, stress, anger, sleep disturbances, and harmful, addictive, and destructive behaviors emerged (Ahmed et al., 2020; Atalan, 2020; Bäuerle et al., 2020; Dozois, 2020). Several additional consequences were also observed following the lockdown interventions, such as fear of contagion, experiences of frustration, social disconnection as well as stigmatization (Brooks et al., 2020; Planchuelo-Gómez, Odriozola-González, Irurtia, & de Luis-García, 2020).

Notably, the psychological impact appears to be very pervasive and severe. There is a clinically prominent pattern of depression and anxiety in SARS-CoV-2 hospitalized patients, which is related to the severity of the consequences of the infection as well as the length of the hospitalization (Alshareef, Al Zahrani, Alzhrani, Suwaidi, & Alamry, 2020; Cao et al., 2020). Apart from infected individuals, general population may also experience several negative psychological consequences associated with the pandemic and its countermeasures, such as depression, anxiety, panic attacks, reduced sleep quality, increased stress response, psychomotor agitation, psychosis, and suicidal tendencies, as well as an increase in adverse coping responses to stressful situations, such as alcohol and drug use. These symptoms occur more frequently in regions where SARS-CoV-2 infections had a higher incidence and were described overall with a worsening of psychological well-being (Ahmed et al., 2020; Bäuerle et al., 2020; Choi, Hui, & Wan, 2020).

Using internet search terms as a proxy, this study examines the psychological consequences on a European level of SARS-CoV-2 containment measures. The study is based on the assumption that individuals are intrinsically motivated, both physiologically and psychologically, to take individual and social actions to restore their health and/or quality of life. Furthermore, we assume that, when experiencing discomfort of a physiological or psychological nature, they utilize symptomatic search parameters in commonly used Internet search engines to get support. As previous studies, including the work by Ginsberg et al. (2009) have shown, this assumption is neither unreasonable nor unrealistic.

The following study is based on scientometric research. The goal of scientometric research is to evaluate scientific questions by analyzing and presenting the structure and regularities of quantitative changes in information elements using scientific methodology (Qiu, Zhao, Yang, & Dong, 2017). Therefore, our study leans on the analysis of dynamics of Internet search parameters of the most common Internet search engine of possible affective problems linked to the different containment measures (Deci & Ryan, 1985; Tang & Ng, 2006). Consequently, we explore which search parameters associated with affective impairment are most salient across different containment measures.

## Methods

We designed and performed this infodemiological study according to the STROBE criteria (*ST*rengthening the *R*eporting of *OB*servational studies in *E*pidemiology; Elm et al., 2007), at the Medical University of Graz in close collaboration with Fraunhofer Austria Research GmbH and the Graz University of Technology. Following the International Statistical Classification of Diseases and Related Health Problems, search parameters belonging to affective disorders were explored. These search parameters have been translated into the official languages of the examined countries.

**Figure 1.**
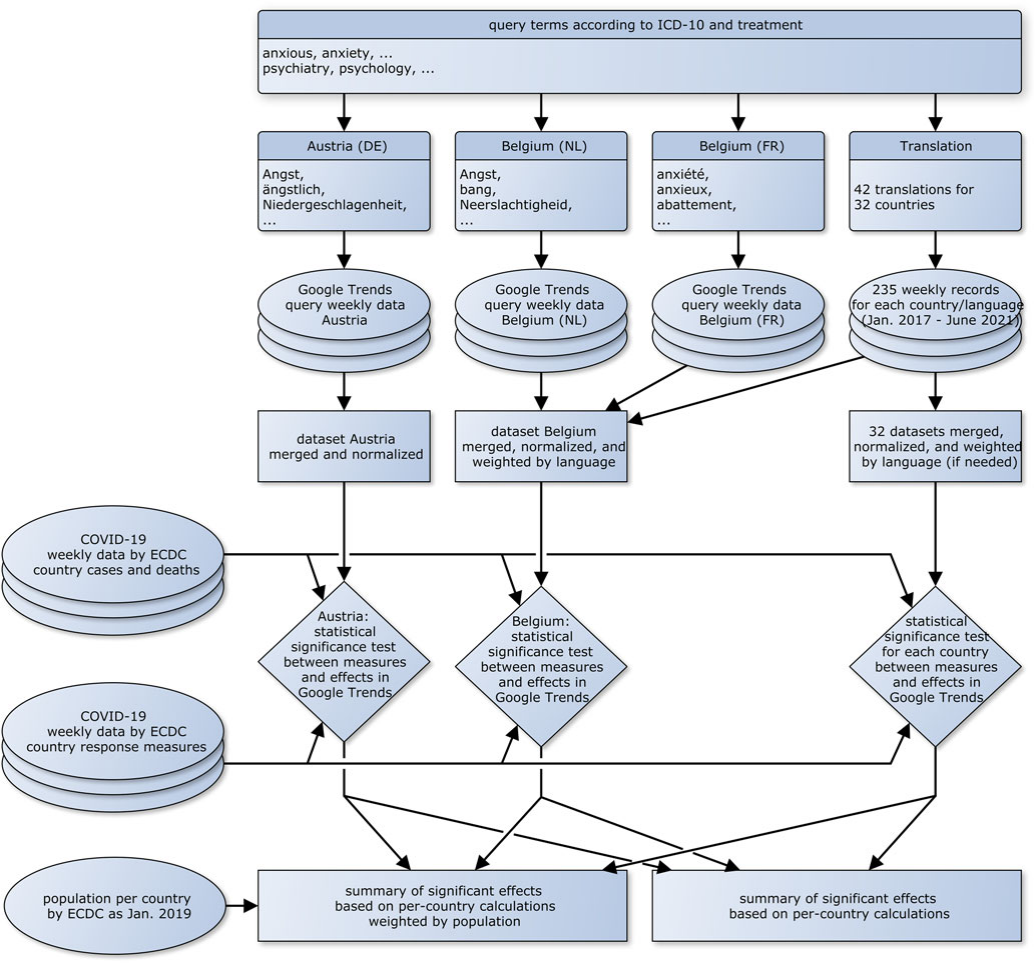
Data flow overview of the process of data acquisition, preparation, and consolidation.

In the period from January 2017 to June 2021, the frequencies of the translated search parameters keywords were acquired using Google Trends analysis and have been have cleansed as described in detail below. These data were temporally and geographically related to the number of cases and countermeasures taken as reported by each country to the European Centre for Disease Prevention and Control (ECDC, 2021). Figure 1 presents the workflow of the data acquisition and analysis of this study. Based on this data and their correlations two main issues of interest are the focus of this study:

- The first research question is to determine which psychological search parameters associated with affective disorders according to the ICD-10 classification are significantly more frequent among the SARS-CoV-2 specific containment measures.
- In the second research question we explore what psychological search parameters concerning psychological support providers occur significantly more often during SARS-Covid2 containment measures.

### Data acquisition and processing

The data set is composed of data provided by the European Centre for Disease Prevention and Control and by Alphabet Inc. (Mountain View, California). The European Centre for Disease Prevention and Control provides the daily number and the 14-day notification rate of new reported COVID-19 cases and deaths by EU/EEA countries. These data provide the basis for describing the course of the pandemic. Furthermore, the European Centre for Disease Prevention and Control provides data on non-pharmaceutical country response measures to SARS-CoV-2 (ECDC, European Centre for Disease Prevention & Control, 2021). The second data source is provided by the holding company Alphabet Inc. offering the Web service Google Trends that analyses the popularity of top search queries in Google Search across various regions and languages via https://trends.google.com/ (Yossi, 2012).

The data set of this study comprehends 235 weekly records from the period January 2017 to June 2021 of 32 European countries with 19 search term categories. The weekly records have been queried covering the following periods:

- January 2017 to June 2021, as well as the nine-months-periods
- January 2017 to September 2017,
- October 2017 to June 2018,
- July 2018 to March 2019,
- April 2019 to December 2019,
- January 2020 to September 2020,
- October 2020 to June 2021.

The goal of splitting the overall period of January 2017 to June 2021 into short intervals of nine months each is to achieve a higher accuracy by avoiding unadjusted normalization and discretization in periods with only few Google queries. Furthermore, to reduce sampling artifacts caused by the daily sampling of the data set of all Google queries, the Google Trends analyses were repeated multiple times on different days (as of December 2021, nine repetitions starting in July 2021).

Each Google Trends analysis has been conducted in all the following 32 countries in all official languages of the corresponding country:

*Austria, Belgium, Bulgaria*, Croatia, Cyprus, Czechia, Denmark, Estonia, Finland, France, Germany, Greece, Hungary, Iceland, Ireland, Italy, Latvia, Lichtenstein, Lithuania, Luxembourg, Malta, Netherlands, Norway, Poland, Portugal, Romania, Slovakia, Slovenia, Spain, Sweden, Switzerland, United Kingdom

i.e. in total, the analyses were performed in 42 individual country-language combinations. Each analysis had a specific, psychological search term in focus, which was manually translated into the respective language. In cases where the translation was ambiguous due to synonyms, declension, etc. all translation variants were used.

The search terms derived from ICD-10 categorizing affective disorders, include:

*anxious, anxiety*, dejection, depressed, depression, exhausted, exhaustion, guilt, insomnia, listlessness, loss of appetite, loss of libido, depressed/sad/ bad mood, panic attack, sad, sadness, sleepless, sleep disorder/problem, tired, weariness, worthless, worthlessness, as well as psychiatry, psychology, psychologist, psychotherapist, psychotherapy, psychological treatment.

In total, depending on the number of synonyms and declensions, up to 38 different search queries are evaluated for each of the 42 individual country-language combinations. Online Supplementary Appendix B comprehends all query terms; both the English ones and their translations. According to Alphabet Inc., Google Trends provides access to queries. This database has been accessed automatically via the official Web-based interface using the Python library ‘pytrends’. The complete source code of the Python scripts to access Google Trends data is listed in online Supplementary Appendix C. The returned search statistics are normalized to the time and location of a query scaled on a range of 0 to 100 based on a topic's proportion to all searches on all topics and rounded to an integer. Additionally, to the introduced rounding error, the dataset returned by Google Trends has a sampling error (Steegmans, 2021). The sampling error occurs because Google uses only a sample of all searches and information on the sample size is not disclosed; and Google Trends uses a different sample on every single day. In particular, the sample size is not adjusted to the distribution of search queries, i.e. the number of samples is not extended for infrequent search queries. In order to overcome these sampling artifacts and the discretization during the internal normalization by Google Trends the downloaded data has to be consolidated to a consistent data set. This process is performed independently for each country and for each search term group.

The first data processing step merges the time series covering the complete analysis period (January 2017 – June 2021) for a specific country and a specific search term group. According to Alphabet Inc. each time series is based on a sampling of all search queries, where the sampling size as well as the size of the underlying total data set is not mentioned, filtered by country and time frame. Then for each week within the time frame the relative proportion of analyzed search terms in the filtered sample is determined, normalized/scaled to 100 and rounded to integer values. Since the sampling of the underlying total data set is done on a daily basis, Google Trends queries will produce different results on different days. The first step merges these different results and determines a weekly average. Afterwards the averaged time series is scaled to 100.0 (but not rounded). In addition to the time series covering the complete analysis period (January 2017 – June 2021) supplementary time series, each covering nine months, increase the overall accuracy. For this purpose, all-time series of a 9-month interval are combined to a short-term, mean time series. A scaling factor is then determined for each 9-month interval so that the maximum of the averaged 9-month interval is mapped to the maximum of the global, averaged time series from the corresponding time interval. In the next processing step the scaled, short-term analysis data is merged. Two reasons for merging the data are distinguished: multilingualism and linguistic variety. Data from countries with more than one official language are queried in all official languages and are merged with weighing factors representing the proportion of corresponding native speakers in the population of the country. This weighted summation is performed in the following countries:

Belgium, Cyprus, Finland, Ireland, Luxembourg, Malta, and Switzerland.

Belgium has three official languages: Dutch, French and German. As no census exists, there are no official statistical data regarding the distribution or usage of Belgium's three official languages; the estimated distributions of primary languages in Belgium (and the used weights) are 59% Dutch, 40% French, and 1% German. Cyprus has two official languages, Greek and Turkish. Based on the 2011 census the distribution (Government of Cyprus, Statistical Service Cyprus, 2022) of native speakers is 81% (Greek) to 1% (Turkish); the completing percentages do not speak any official language natively and the resulting weights are 0.988 and 0.012. The two main official languages of Finland are Finnish and Swedish. According to official statistics Finnish is spoken by 87% (weighting factor 0.946) of the population, and Swedish is spoken by 5% (weighting factor 0.054) of the population (Statistics Finland, 2021). In the Republic of Ireland, the languages Irish and English have official status; according to the latest Europe-wide survey of languages in Europe by the European Commission 93% of the population speak English and 3% of the population speak Irish as mother language (Van Parys, 2012); the corresponding weights are 0.969 and 0.031. The three official languages of Luxembourg are French, German and Luxembourgish. In the private sector the main language is French (56%; 0.683), followed by Luxembourgish (20%; 0.244), and German (6%; 0.073) (Pigeron-Piroth & Fehlen, 2015). Malta has two official languages: Maltese and English. A study collecting public opinion on what language was ‘preferred’ discovered that 86% (weighting factor 0.878) of the population express a preference for Maltese and 12% (weighting factor 0.122) for English (Capdevila, 2004). Switzerland has four national languages: mainly German (spoken by 62.8% of the population in 2016), French (22.9%), Italian (8.2%), and Romansh (0.5%) (Confederatione Svizerra, Federal Statistical Office, 2021). Linguistic varieties due to synonyms within one language are averaged without weighting. Last but not least, a comprehensive dataset was created combining all countries weighted by population size as currently listed by the ECDC (as of July 2021).

**Figure 2.**
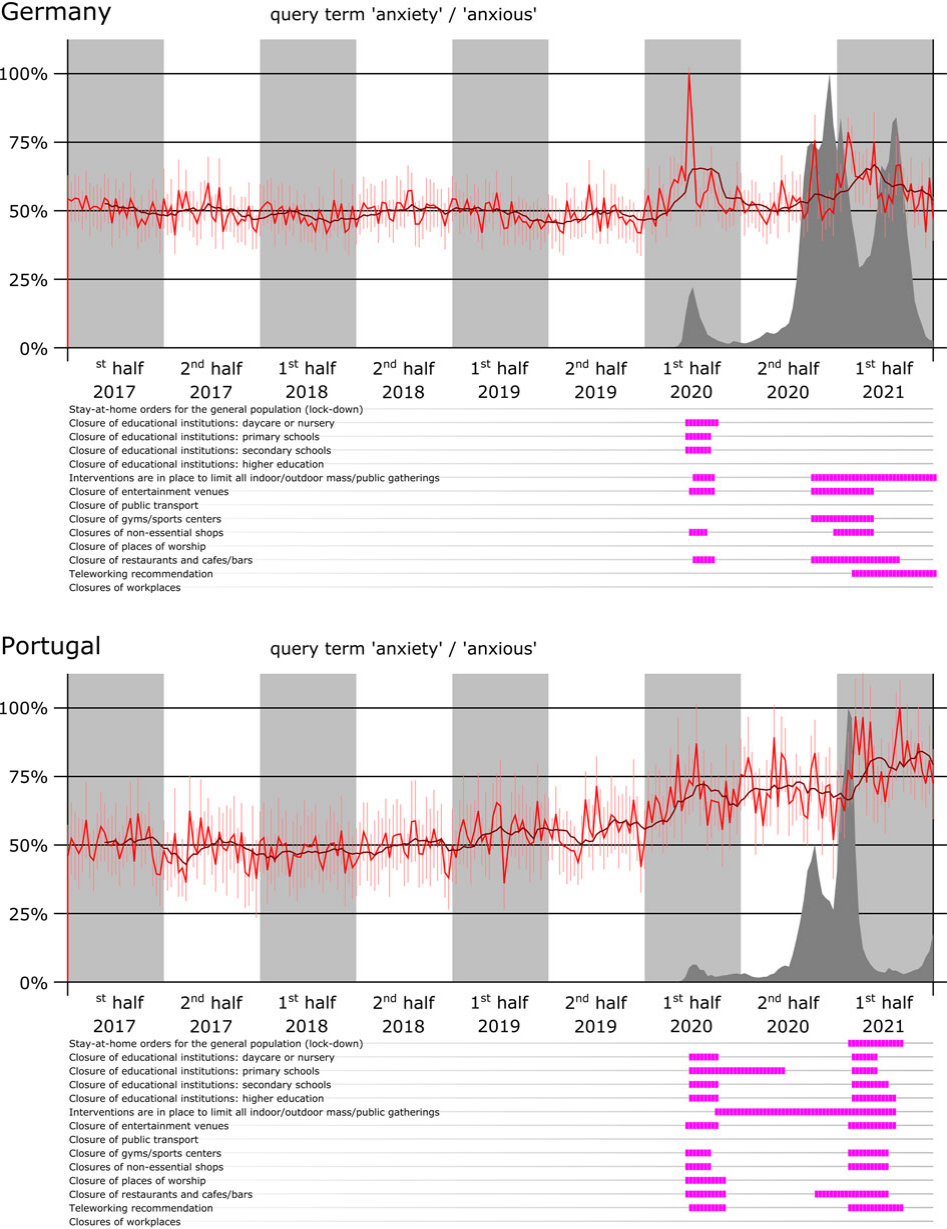
The data acquisition provides a time series for each country and each search term. The upper visualization shows the data on “anxiety/anxious” searches in Germany; the lower one accordingly for Portugal. Each graph shows in red the relative number of searches scaled to 100%, with the uncertainty due to sampling and rounding plotted as the estimated standard deviation for each time point as an additional thin red line; the black line shows the moving average of a two-month time window. The temporal relation to the pandemic is based on the number of nationally reported infection cases, which is also scaled and shown as a dark gray plot. The counter measures in force at the national level at that time are plotted below the time series.

The results of the data acquisition are a time series for each pair of country and query term category. Figure 2 shows as examples the time series for search queries for the term ‘anxiety/anxious’ from the countries Germany (top) and Portugal (bottom). These diagrams illustrate two patterns that can be observed in several data sets: (1) the time series plot of the term ‘anxiety/anxious’ in Germany shows a peak in temporal proximity to a pandemic wave; (2) the graph of the Portuguese data set shows a gradually increasing trend with the beginning of the pandemic. This trend is particularly well visible in the two-month moving average window.

### Data analysis

The main analysis step is based on Mann–Whitney-U-tests carried out using IBM-SPSS 22 (Armonk, New York). With this test, we compare for each country the frequency of a query parameter with respect to the time in which a countermeasure was taken and implemented. For each triplet of country, term, and countermeasure, the test either returns *invalid* (because the measure was not implemented in the corresponding country, or Google Trends returns an empty time series), or a *p*-value. If the test returns *p*-values less than or equal to 0.05, the result is considered significant, and a value less than 0.01 is considered very significant, i.e. in these cases the distributions in the two groups – before and during the countermeasure – differed (very) significantly.

All results for all triplets of country, term, and countermeasure are listed in online Supplementary Appendix A. Based on the identification of the significant changes, the dependency on the individual countries is eliminated in the second step: This is done by determining the percentage of countries in which a countermeasure led to a significant or a very significant change. This second step considers all countries equally – regardless of their population size. As the European countries differ in population size significantly (see Table 1), it is sensible to take this aspect into account. To give more weight to population size, the second step was repeated in a third step with a weighting by population size, i.e. for a countermeasure and for a search term, the weighted percentage is determined rather than the simple percentage of affected countries with (very) significant changes. The used weights correspond to the population size as currently listed by the ECDC (see Table 1).

**Table 1.** Each European country implemented countermeasures independently (Yan et al., 2021)

Country	Population	Accumulated countermeasure days
Austria	8 901 064	6477
Belgium	11 522 440	7113
Bulgaria	6 951 482	4401
Croatia	4 058 165	5718
Cyprus	888 005	5242
Czechia	10 693 939	7228
Denmark	5 822 763	6122
Estonia	1 328 976	5704
Finland	5 525 292	5185
France	67 320 216	6023
Germany	83 166 711	6500
Greece	10 718 565	6586
Hungary	9 769 526	4579
Iceland	364 134	4233
Ireland	4 964 440	7596
Italy	59 641 488	6602
Latvia	1 907 675	6591
Liechtenstein	38 747	4428
Lithuania	2 794 090	6859
Luxembourg	626 108	6163
Malta	514 564	5832
Netherlands	17 407 585	7290
Norway	5 367 580	5276
Poland	37 958 138	8197
Portugal	10 295 909	6351
Romania	19 328 838	7934
Slovakia	5 457 873	5888
Slovenia	2 095 861	5463
Spain	47 332 614	6349
Sweden	10 327 589	4676
Switzerland	8 606 033	5367
United Kingdom	68 059 863	3553
Minimum ↔ Maximum	38 747 ↔ 83 166 711	3553 ↔ 8197
Average ± Standarddeviation	16 554 884 ± 22 556 046	5985 ± 1099

This table lists the population size for each country, and the accumulated product of each countermeasure with the period of its duration (measured in days). All data are provided by the European Centre for Disease Prevention and Control (ECDC, 2021)

Finally, in a last step the dependency on the search term is eliminated by determining the percentage of countries in which a countermeasure led to a significant or a very significant change in any search term. Analog to the second step, this step is performed a second time using the population weight.

## Results

Analyzed by country, the following significant differences of psychological aspects related to the containment measures were found: the countermeasure ‘teleworking recommendations’ is associated with significant or very significant changes for the search term ‘anxiety’ in 17 countries including Austria, Belgium, Croatia, France, Germany, Greece, Ireland, Italy, Luxembourg, Malta, Norway, Poland, Portugal, Romania, Slovenia, Spain, and Switzerland. No significant changes have been found in 10 countries: Bulgaria, Czechia, Denmark, Finland, Latvia, Lithuania, Netherlands, Slovakia, Sweden, and United Kingdom. For the pair ‘teleworking recommendations’ and ‘anxiety’ has a significant change in 17 of 27, or 63% of the countries. Weighted by population size, this value even rises to 73.9%. If this calculation is performed with ‘teleworking recommendations’ not only for all countries, but also for all search terms examined, significant changes are identified in 37.5% of all countries and all search terms. Table 2 lists all results of these calculations and explicitly names the first and second dominating effect.

**Table 2.** Each countermeasure is tested statistically on its influence on Internet search terms of psychological disorders and symptoms

Measure	Influence by country (%)	1st dominating effect by country	2nd dominating effect by country
Teleworking recommendations	37.5	Anxiety	Worthlessness
Interventions are in place to limit all indoor-outdoor mass public gatherings	36.9	Anxiety	Depression
Closure of entertainment venues	36.9	Anxiety	Depression
Closure of gyms sports centers	33.9	Anxiety	Panic attack
Closure of workplaces	33.3	–	–
Closure of educational institutions: higher education	31.3	Depression	Sadness
Closure of educational institutions: daycare	31.1	Sadness	Anxiety
Closure of restaurants and cafes	30.9	Insomnia	Anxiety
Closure of educational institutions: secondary schools	30.5	Depression	Anxiety
Closure of educational institutions: primary schools	28.9	Anxiety	Depression
Stay at home orders for the general population (lock-down)	28.8	Anxiety	Depression
Closure of non-essential shops	27.9	Depressed mood	Panic attack
Closure of places of worship	26.1	Depression	Anxiety
Stay at home recommendation	15.9	Anxiety	Sadness
Closure of public transport	5.3	Depression	–

The table is ordered by the percentage of countries with a (highly) significant correlation. The search term with the highest correlation is listed as dominating effect.

To give more weight to population size, this calculation has been repeated with a weighting by population size. Weighted by population, among examined countries leads to different results due to the large variation in European population sizes as listed in Table 1. In the weighted calculation, the following significant differences of psychological aspects related to the containment measures have been identified: the ‘closure of workplace’ has a significant / very significant effect on 57% of the population, if in place. The measure is correlated with ‘listlessness’ and ‘panic attacks’; the ‘closure of entertainment venues’ is a countermeasure which is correlated with ‘worthlessness’ and ‘anxiety’ for 50.4% of the population of all examined countries. A similar influence can be measured for the ‘closure of gyms sports centres’. In times, in which this measure is in place, 50% of the population shows a significant change in the frequencies of the search terms ‘anxiety’ and ‘panic attacks’. The complete list of measures, the proportion of the population showing significant changes in the frequencies of the analyzed query terms, and the terms with the highest change rate are listed in Table 3.

**Table 3.** Analogous to Table 2, each countermeasure is tested statistically on its influence on Internet search terms of psychological disorders and symptoms

Measure	Influence by population (%)	1st dominating effect by population	2nd dominating effect by population
Closure of workplaces	57.6	Listlessness	Panic attack
Closure of entertainment venues	50.4	Worthlessness	Anxiety
Closure of gyms sports centers	50.0	Anxiety	Panic attack
Closure of educational institutions: higher education	47.3	Sadness	Depression
Closure of non-essential shops	46.2	Panic attack	Anxiety
Teleworking recommendations	45.7	Anxiety	Worthlessness
Closure of educational institutions: daycare	45.1	Worthlessness	Anxiety
Interventions are in place to limit all indoor-outdoor mass public gatherings	45.1	Panic attack	Anxiety
Stay at home orders for the general population (lock-down)	44.6	Sadness	Worthlessness
Closure of educational institutions: secondary schools	43.1	Depression	Anxiety
Closure of places of worship	40.4	Depression	Sadness
Closure of restaurants and cafes	39.7	Worthlessness	Anxiety
Closure of educational institutions: primary schools	38.9	Anxiety	Depression
Stay at home recommendation	14.8	Anxiety	Weariness
Closure of public transport	6.8	Depression	–

In this table the results are weighted with the population size of each correlated country.

Finally, all data sets have been combined into two European datasets: a dataset that considers all countries equally, regardless of population size, and a weighted dataset by population size. Both data sets reveal significant increases in the frequencies of most search terms related to psychological aspects in the time ranging three years before and since the official pandemic declaration by the WHO. In detail, the Internet search terms ‘anxiety’, ‘dejection’, ‘weariness’, ‘listlessness’, ‘loss of appetite’, ‘loss of libido’, ‘panic attack’, and ‘worthlessness’ have been entered in search queries more often in Europe during the pandemic than before. Furthermore, the non-weighted dataset shows an increase in ‘insomnia’ and the population-weighted dataset shows an increase in ‘exhaustion’ and in ‘guilt’; however, these effects only show up in one data set – not in both.

In contrast, a significant decrease in the search frequency could be found for the search term ‘depression’ in the non-weighted dataset; weighted by population there is no significant increase or decrease for the search term ‘depression’ (see Table 4).

**Table 4.** The overall impact of the pandemic on individual search parameters before and during the pandemic in Europe

	Changes in psychological search parameters (*p-*Value) before and after the confirmed SARS-CoV-2 pandemic by the WHO.	
Psychological aspect of an affective disorder	Independent of population size	Weighted by population size
Anxiety	<0.001^a^	<0.001^a^
Dejection	<0.001^a^	<0.001^a^
Depression	<0.001^b^	0.55^c^
Depressed mood	0.93^c^	0.48^c^
Exhaustion	0.25^c^	<0.019^a^
Weariness	<0.001^a^	<0.001^a^
Listlessness	<0.001^a^	<0.001^a^
Loss of appetite	<0.001^a^	<0.001^a^
Loss of libido	<0.001^a^	<0.001^a^
Panic attack	<0.001^a^	<0.001^a^
Sadness	0.19^c^	0.48^c^
Bad mood	0.53^c^	0.33^c^
Insomnia	<0.019^a^	0.24^c^
Sleeping problem	0.79^c^	0.20^c^
Worthlessness	<0.001^a^	<0.001^a^
Guilt	0.07^c^	<0.006^a^
Treatment or treatment provider	Changes in treatment or treatment provider related search parameters (*p*-Value) before and after the confirmed SARS-CoV-2 pandemic by the WHO.	
	Independent of population size	Weighted by population size
Psychology	<0.017^b^	0.35^c^
Psychiatry	0.51^c^	0.75^c^
Psychologist	<0.001^a^	<0.001^a^
Psychotherapist	<0.001^a^	<0.001^a^
Psychotherapy	<0.001^a^	<0.002^a^
Psychological treatment	<0.037^a^	0.72^c^

a Changes of the psychological-related search parameter **increased** significantly after the first confirmed COVID-19 case was reported.

b Changes of the psychological-related search parameter **decreased** significantly after the first confirmed COVID-19 case was reported.

c No significant changes.

**Figure 3.**
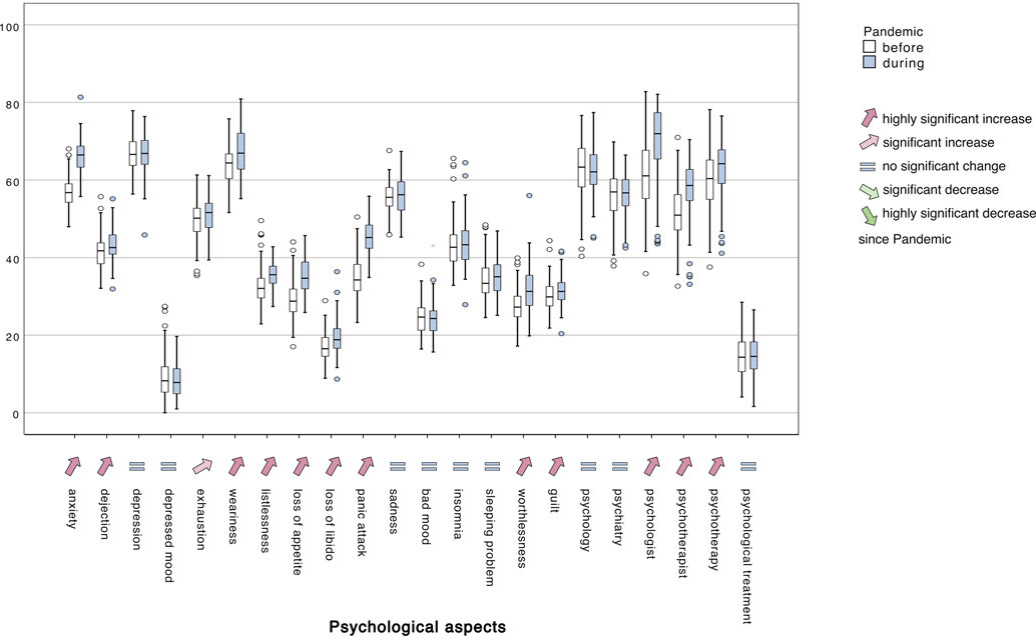
The overall distribution of frequencies in the population-weighted data set of all countries (in a range between 0 and 100) of most search terms related to psychological aspects in the time ranging three years before and since the official pandemic declaration by the WHO.

Considering the frequency of search criteria related to treatment or treatment provider, both datasets show that the search frequency has increased significantly since the SARS-CoV-2 pandemic for the following search terms: ‘psychologist’, ‘psychotherapist’, ‘psychotherapy’. A significant decrease occurred only for the search term ‘psychology’. As with the search query on ‘depression’, a significant increase for ‘psychology’ is only found in the unweighted dataset; the weighted dataset shows no significant increase or decrease for ‘depression’ and ‘psychology’. All results of the complete European data set are listed in Table 4 and plotted in Fig. 3.

## Discussion

To our knowledge, this is the first study that informetrically examines psychological consequences of the SARS-CoV-2 pandemic of respective containment measures within 32 countries.

The curated dataset used has several characteristic differences compared to other existing datasets: The dataset is focused on psychological aspects and does not address physical symptoms in comparison to Szilagyi et al. (2021). Moreover, the curated dataset has been designed to be very comprehensive in time, i.e. with a pre-pandemic period of almost three years, it is possible to extract a pre-pandemic model based on data and compare it with the pandemic data. In this way, temporal differentials can be identified (compared to ‘Google COVID-19 Search Trends symptoms dataset’, Google LLC, 2020). Furthermore, the dataset includes a regionally differentiated picture that is not otherwise available for Europe in this precision (data.world, 2022).

The primary goal this study is to examine the psychological impact of each containment measure, through analysis of psychological Internet query terms temporarily related to the corresponding countermeasures. As a second objective, this study determines the trends in search parameters related to psychological and psychiatric treatment and treatment providers.

Our results show that the search criteria for all psychological aspects that constitute different symptoms of affective-psychological disorders according to ICD-10 have changed considerably since the pandemic; see Table 4 and Fig. 3 for details. Exploring the data by population, the 32 countries examined reveal that with the introduction of containment measures, in particular the following psychology related search terms have changed significantly:

‘anxiety’, ‘dejection’, ‘listlessness’, ‘loss of appetite’, ‘loss of libido’, ‘panic attack’, ‘weariness’, and ‘worthlessness’

### Anxiety

The very prominent psychological aspect ‘anxiety’ involves a feeling that emerges in response to an unspecified threat. It manifests itself not only in a sensation of threat of some kind, but it is also commonly accompanied by a large cascade of physiological reactions, such as changes in muscle tension, heart rate variability, or blood pressure, among others. It induces behavior of avoidance and reinforces unfavorable coping patterns. Experienced frequently, anxiety can adversely affect quality of life and, in further consequence, cause psychological as well as physiological illnesses. According to our results, the feeling of anxiety, is highly prominent in the pandemic period and ranks highest in most of the containment measures, such as: ‘closure of educational institutions: primary schools’, ‘closure of entertainment venues’, ‘closure of gyms sports centres’, ‘interventions are in place to limit all indoor-outdoor mass public gatherings’, ‘stay at home orders for the general population (lock-down)’, ‘stay at home recommendation’ and ‘teleworking recommendations’ (see Tables 2 and 3).

Three main mechanisms can be attributed for the high level of anxiety associated with the SARS-CoV-2 pandemic outbreak and its containment measures: the evaluation of the current situation and resulting responses, the concern over infection and its health consequences, and the impact of the stream of concerning information provided by the media.

Two basic models, cognitive emotion theory and evaluation theory, originate from emotion psychology can be referred to in the explanation of the affective consequences of the pandemic and the containment measures. Both theories assume that an event is evaluated according to its actuality, value, as well as its threatening nature. As a result, such an evaluation triggers emotions, impulses to take certain actions, and physiological reactions (Arnold, 1950; Lazarus, 1991). Transferred to the pandemic situation, this model would result in greater anxiety, since the virus infection and its health consequences might be evaluated by individuals as very threatening. The containment measures, such as the closure of schools, various workplaces, or areas of social activities, leads to a suppression of impulses for actions, such as visiting loved ones, pursuing social activities, working, or going to school, all of which can be perceived as a threat and an interference. Consequently, higher anxiety occurs at the level of perception. Anxiety during the pandemic can be attributed also to specific needs in interpersonal relationships and the evaluation and impact of specific situations. In addition, the social psychological model Construal Level Theory (CL theory) offers another possible explanation for the interpersonal experiences related to the pandemic and lockdown events. The CL theory provides an explanation of how we mentally evaluate individuals, objects, events, and ideas that we encounter, a process that is directly dependent by our psychological distance from the respective entities. However, psychological distance varies from person to person and is determined by geographic circumstances, time-related determinants, social acquaintances, and the probability of events occurring (Bowen, 2021; Stillman, Fujita, Sheldon, & Trope, 2018; Zheng et al., 2020). In accordance with CL theory, a prominent influence in the pandemic is shown by geographical distances, among others to loved ones, friends to beloved activities, such as sports or other types of hobbies, but also to regular structured activities, such as work or school visits. The temporal aspect of assessing the pandemic situation is, due to the high number of infections, recurrent containment measures, and constantly new virus mutations, a great unknown that presumably may trigger insecurity. The strong restriction of social interactions as well as the estimation of a possible high occurrence of infection as well as associated consequences cover all levels of a psychological distance, whose evaluation and individual processing might cause affective reactions such as anxiety (Bowen, 2021; Zheng et al., 2020).

Another recent fear construct linked to the pandemic is coronaphobia, which can cause both functional and psychological problems. It can be defined by the worry of infecting oneself or family members with the virus SARS-CoV-2 (Vizard, Davis, White, & Beynon, 2020). However, it cannot be ruled out that coronaphobia may also cause health anxiety due to a high degree of misinterpretation of bodily sensations, so that harmless physiological sensations or changes were interpreted as signs of infection. In addition, severe health anxiety ensured that many people practiced extensive safety behaviors in the aftermath of the pandemic, such as excessive hand hygiene, frequently laundering, and over-disinfection of hands or clothing. The misinterpretation of physical sensations during the pandemic may have led to an increased anxiety regarding the possibility of suffering from a covid infection (Asmundson & Taylor, 2020).

Lastly, the dissemination of information may have contributed to the increase or development of anxiety about the pandemic and its containment efforts. The rapid availability of information, the constantly increasing number of infections, the details about the virus’ fast spread, the many fatalities, the initially unclear treatment methods, and the widely strict – and also diverse and common – containment measures resulted in a large amount of predominantly negative reports about the virus from almost all media sources, which could ultimately have an unfavorable impact on health anxiety (King et al., 2022; Landi, Pakenham, Boccolini, Grandi, & Tossani, 2020) and furthermore on the frequency of Google queries for parameters such as ‘anxiety’.

### Panic attack

Based on the data acquired in our study, panic attacks are also among the psychological aspects that are listed particularly frequently and are associated with containment measures. They are characterized among others with symptoms such as: chest pain, shortness of breath, generalized anxiety, coronaphobia, tremor, dizziness, nausea, vomiting (Bhatia, Goyal, Singh, & Daral, 2020; Javelot & Weiner, 2020). In the following containment measures, the search parameter ‘panic attack’ features prominently: ‘closure of non-essential stores’, ‘interventions are in place to limit all indoor-outdoor mass public gatherings’, ‘closure of gyms sports centres’ and ‘closure of workplaces’. Various explanatory models for panic disorder can be derived: among others, panic attacks can also be explained by fear of fear, as an interoceptive conditioning, whereby a constant attentiveness to bodily functions is derived.

In the pandemic situation, it can also be assumed that the evaluation of actual situation and the associated containment measures may play a substantial part in the development of panic reactions. Assessing the current situation as extremely threatening and abstract due to the mobility restrictions, the obligatory stay at home and the impulses of not being able to satisfy the own needs can subsequently lead to panic states. It has been shown that mental health status or mental coping during the pandemic depends significantly not only on numerous factors such as sociodemographic characteristics like social class, ethnicity, and gender, but also on mental coping measures. People who are unable to adequately manage psychological stress are not only more vulnerable to affective disorders such as anxiety or panic attacks but are also at higher risk of developing physically linked diseases as well. Therefore, containment measures such as closing stores, gyms, sports centers, workplaces and restricting mass public gatherings, while protective against infectious diseases, may result in highly negative psychological consequences (Ellwardt & Präg, 2021). Conspiracy theories in the media, rumors of increased containment measures, and rapidly circulating images of sick or dying people on social media fuel consequences such as panic attacks and anxiety (Saadat et al., 2020; The Lancet, 2020; Yoon, Feyissa, & Suk, 2021). While for some people the consequences of negative information trigger a brief emotional response latency, a weak information filter allows for a fear response to panic reactions that are sustained by new negative information (Bushuyev, Bushuieva, Onyshchenko, & Bondar, 2021; Sharma et al., 2020).

### Depression and worthlessness

Considering the general trends in search parameters only for the period before and after the outbreak of the pandemic, our results show significant changes in the frequency of the search term ‘depression’ in the unweighted dataset and no significant changes in the weighted dataset. A depression refers to a mental illness characterized by dejection, loss of interest, exhaustion, and listlessness. It is accompanied by other secondary symptoms, such as feelings of guilt, lack of concentration, self-doubt, or insomnia. Severe courses of this disease can lead to suicide. The causes are genetic-physiological factors, as well as psychosocial strain, stress load or a combination of these.

Contrary to expectations, our study shows a significant decrease in the frequency of the search term ‘depression’ since the pandemic outbreak in at least one dataset. In studies assessing associations between a pandemic and depression, an increase in depression rates was found following a pandemic outbreak. Thus, in a 2021 study, it was found that after the spread of COVID-19 in Wuhan, one in five (19.2%) suffered from moderate to severe depression (Zhong, Huang, & Liu, 2021). A representative survey in the United Kingdom found that the pandemic significantly increased the number of people with a depressive disorder, identifying the following risk factors as favoring the depressive disorder development during a pandemic: female gender, younger age, socioeconomic problems and disabled individuals (Vizard et al., 2020). A meta-analysis shows a world-wide 7-fold increase in the prevalence of depression since the pandemic outbreak (Bueno-Notivol et al., 2021). Of 15 000 people examined who had been in quarantine for 10 days, 29% showed post-traumatic stress disorder and 31% experienced symptoms of depression (Huremović, 2019).

It is not explainable with the previous theoretical concepts, however, why our result does not show the expected trend. A possible explanation may be found in the variability of languages; as symptoms increase or decrease, other languages may use different search terms whose nuances are lost in translation. Online Supplementary Appendix B lists up to 4 different translations each for the noun stems depression (depressive, etc.) and sadness (sad, etc.). Furthermore, it cannot be ruled out whether depressed individuals show different online search behavior due to a pandemic than pre-pandemic, whether depression prevented them from conducting ‘online searches’, nor whether multiple factors may have influenced this outcome. Additional studies are needed to investigate this particular trend.

Our study further shows that the search term ‘depression’ appeared as a search parameter in the following containment measures: ‘interventions in place to limit all indoor-outdoor mass public gatherings’, ‘closure of entertainment venues’, ‘closure of educational institutions: higher education’, ‘closure of educational institutions: secondary schools’, ‘closure of educational institutions: primary schools’, ‘stay at home orders for the general population (lock-down)’, ‘closure of places of worship’, ‘closure of public transport’.

A component of depression, the feeling of worthlessness, emerged at second position, being the most frequently associated search term among the different measurement procedures. Depressed people often experience feelings of worthlessness and guilt. Individuals are quick to assign unpleasant incidents to themselves and blame themselves, even if these feelings are of an irrational nature. The feeling of worthlessness as a search parameter emerged in the following containment measures: ‘closure of entertainment venues’, ‘teleworking recommendations’, ‘closure of institutions: daycare’, ‘stay at home orders for the general population (lock-down)’, ‘closure of restaurants and cafes’.

The strong prominence of the psychological terms ‘depression’ and ‘worthlessness’ that accompanied the above-mentioned containment measures during the pandemic can be attributed to several possible explanations. One of these involves the fact that the pandemic appears to have altered the entire social structure worldwide, from employment status to educational situation to outdoor activities (Foley & Cooper, 2021). Containment is a very efficient way of interrupting chains of infection, as they separate infected persons from non-infected ones. Not being able to deal with everyday matters, experiencing dramatic events such as the loss of a loved one or an acquaintance, or constant negative news about deaths or numbers of infected person, can lead to increased anxiety in isolation as well as in a pandemic, and can further trigger serious illnesses such as post-traumatic stress disorder or depression. Studies show that younger people were severely affected psychologically by the pandemic and the lockdown measures. As a result, children and adolescents lost the opportunity to participate in school or extracurricular activities, which gave them a sense of structure or daily rhythm (Courtney, Watson, Battaglia, Mulsant, & Szatmari, 2020; Davy et al., 2021; Wang et al., 2020). Furthermore, because of the pandemic and following the lockdown measures, people faced restrictions in their daily activities, which were likely associated with insecurity and a sense of threat. Also, during the pandemic, many people had experiences that threatened their livelihoods, such as losing their jobs, which had economic, social, and ultimately life consequences (Kaushik & Guleria, 2020).

In the context of depressive consequences of the pandemic, social networks and media may also play another major role. The information contained in these areas can be a useful tool for gaining more knowledge about pathogens and treatment methods, while on the other hand, they provide a breeding ground for health impairment (Zhong et al., 2021).

### Looking for psychological support options

Considering searches of support for a possible psychological burden, illness, or disorder, analyses were also conducted in our study. We analyzed to what extent the corresponding search terms have changed significantly since the pandemic. Among them, our results revealed that the search parameters ‘psychologist’, ‘psychotherapist’, ‘psychotherapy’ have increased highly significantly (*p* < 0.01) since the COVID-19 pandemic.

As a result of the psychological distress caused by the pandemic, we assume that people are significantly more likely to search for psychological and psychotherapeutic support on the Internet.

The more general search term ‘psychology’ became significantly less frequently searched since the pandemic (*p* < 0.05) in the unweighted dataset. However, there is a possible interpretation that this search query refers to more general terminology such as psychology studies, psychology books or other general interests in the field of ‘psychology’ that have received less interest since the pandemic outbreak and its containment measures.

## Limitations

The individual data sources have some limitations. The data on non-pharmaceutical interventions and response measures have been collected by the European Centre for Disease Prevention and Control. The European Centre for Disease Prevention and Control uses multiple information sources per country such as Ministries of Health or National Public Health Institutes, and the obtained data is systematically cross checked with data from the World Health Organization. The limitations of this data source are described in detail by the ECDC (2021).

The data provided by Google Trends have a sampling error (Steegmans, 2021) which has been compensated by the data acquisition strategy implemented in this study. However, there is certainly an interpretive limitation: searching for a term on the Internet does not and should not equate to a diagnosis of a given condition; nevertheless, previous studies showed connections and correlations between the users’ Internet queries and their well-being (Ginsberg et al., 2009).

Nevertheless, it must be clearly emphasized that (a) the reach of search engines is limited in market coverage and therefore they are probably not representative and (b) intentions are not stored for the search queries; for example, someone who googles psychology may be looking for psychological help, or looking for information on studying psychology, or may simply not know the word. All these intentions lead to the same search query and cannot simply be traced back to the corresponding reason.

## Conclusions

Our study uses trends in the Internet search parameters of psychological aspects according to the ICD-10 classification in order to determine how they changed during the pandemic due to COVID-19 and the containment measures. For this purpose, a comprehensive dataset was compiled to identify links and significant correlations. As a result, the study reveals three psychological aspects extremely prominent due to the pandemic and its containment measures: anxiety, worthlessness, and panic attack. These three psychological aspects vary in severity depending on the measures implemented.

Furthermore, the results show that the frequency of seeking psychological support measures has increased significantly since the pandemic.

## Supporting information

Szilagyi et al. supplementary material 1 Szilagyi et al. supplementary material

Szilagyi et al. supplementary material 2 Szilagyi et al. supplementary material

Szilagyi et al. supplementary material 3 Szilagyi et al. supplementary material

## Supplementary material

The supplementary material for this article can be found at https://doi.org/10.1017/S0033291723001563.
