# Supplementary material for: Impact of the pandemic and its containment measures in Europe upon aspects of affective impairments: a Google Trends informetrics study
Source: Psychol Med. 2023 Jun 26;53(16):7685–97. doi: 10.1017/S0033291723001563 (PMC10755220; doi:10.1017/S0033291723001563)
Supplement: Szilagyi et al. supplementary material 1 — Szilagyi et al. supplementary material [file S0033291723001563sup001.pdf]

Measure	Effect	Austria 8.901.064	Belgium 11.522.440	Bulgaria 6.951.482	Croatia 4.058.165	Cyprus 888.005	Czechia 10.693.939	Denmark 5.822.763	Estonia 1.328.976	Finland 5.525.292	France 67.320.216	Germany 83.166.711	Greece 10.718.565	Hungary 9.769.526	Iceland 364.134	Ireland 4.964.440	Italy 59.641.488	Latvia 1.907.675	Liechtenstein 38.747	Lithuania 2.794.090	Luxembourg 626.108	Malta 514.564	Netherlands 17.407.585	Norway 5.367.580	Poland 37.958.138	Portugal 10.295.909	Romania 19.328.838	Slovakia 5.457.873	Slovenia 2.095.861	Spain 47.332.614	Sweden 10.327.589	Switzerland 8.606.033	United Kingdom 68.059.863	significant changes	non-significant changes	percentage of countries with significant influence	significant changes weighted by population	non-significant changes weighted by population	percentage of countries with significant influence weighted by population
stay at home recommendation	anxiety				0,141				0,154			0,002	0,422	0,137		0,278	0,616	0,512	0,188	0,122			0,032	0,089	0,004	0,000						0,008	0,836	5	11	31,3%	157.434.376	168.649.115	48,3%
	dejection								0,791			0,385	0,270			0,725	0,353	0,005		0,253			0,050	0,385	0,567	0,548						0,619	0,085	2	11	15,4%	19.315.260	292.901.793	6,2%
	depression				0,040				0,240			0,000	0,215	0,426		0,167	0,125	0,058	0,382	0,001			0,000	0,588	0,177	0,698						0,739	0,085	4	12	25,0%	107.426.551	218.656.940	32,9%
	depressed mood											0,118					0,624																0,376	0	3	0,0%	0	210.868.062	0,0%
	exhaustion				0,029							0,002	0,578	0,316		0,550	0,269			0,890			0,708	0,145	0,966	0,975						0,020	0,323	3	10	23,1%	95.830.909	226.977.184	29,7%
	weariness				0,039				0,849			0,001	0,494	0,302		0,995	0,093	1,000	0,349	0,223			0,122	0,043	0,049	0,287						0,232	0,085	4	12	25,0%	130.550.594	195.532.897	40,0%
	listlessness				0,177				0,054			0,099	0,488	0,761		0,518	0,658			0,875			0,378	0,097	0,052	0,001						0,635	0,488	1	13	7,1%	10.295.909	313.841.160	3,2%
	loss of appetite				0,490							0,343		0,180		0,469	0,096						0,137		0,374	0,877						0,232	0,144	0	10	0,0%	0	303.927.858	0,0%
	loss of libido											0,423				0,653							0,877									0,899	0,216	0	5	0,0%	0	182.204.632	0,0%
	panic attack				0,278				0,639			0,477		0,170		0,498	0,180	0,331		0,018			0,048	0,095	0,412	0,006						0,073	0,087	3	11	21,4%	30.497.584	284.828.595	9,7%
	sadness				0,460				0,623			0,104	0,505	0,275		0,021	0,906	0,778		0,007			0,018	0,221	0,003	0,863						0,711	0,988	4	11	26,7%	63.124.253	262.920.491	19,4%
	bad mood											0,782				0,460	0,735						0,546	0,118	0,388	0,959						0,729	0,108	0	9	0,0%	0	295.467.747	0,0%
	insomnia				0,332				0,050			0,743	0,477	0,295		0,312	0,115	0,378		0,324			0,953	0,014	0,538	0,511						0,005	0,256	3	12	20,0%	15.302.589	310.742.155	4,7%
	sleeping problem				0,202				0,476			0,910	0,592	0,768		0,894	0,616	0,610					0,370	0,943	0,274	0,350						0,134	0,087	0	14	0,0%	0	323.250.654	0,0%
	worthlessness								0,621			0,003		0,520		0,811	0,105						0,198	0,804	0,231	0,635						0,635	0,746	1	10	9,1%	83.166.711	223.399.538	27,1%
	guilt											0,315	0,396	0,166		0,001	0,401						0,815	0,088	0,061	0,803						0,098	0,195	1	10	9,1%	4.964.440	310.991.398	1,6%
	psychology				0,238				0,760			0,001	0,739	0,140		0,117	0,637	0,462	0,404	0,002			0,071	0,223	0,122	0,439						0,929	0,353	2	14	12,5%	85.960.801	240.122.690	26,4%
	psychiatry				0,986				0,095			0,005	0,317	0,153		0,642	0,102	0,314		0,490			0,000	0,183	0,657	0,022						0,812	0,090	3	12	20,0%	110.870.205	215.174.539	34,0%
	psychologist				0,001				0,050			0,000	0,397			0,456	0,426	0,020		0,010			0,017	0,689	0,005	0,021						0,859	0,102	8	6	57,1%	158.917.249	157.357.969	50,2%
	psychotherapist								0,333			0,000		0,384		0,670	0,470	0,962		0,261			0,001	0,905	0,986	0,622						0,672	0,087	2	11	15,4%	100.574.296	210.693.718	32,3%
	psychotherapy				0,245				0,974			0,037	0,858	0,190		0,093	0,825	0,826		0,792			0,000	0,505	0,038	0,233						0,272	0,087	3	12	20,0%	138.532.434	187.512.310	42,5%
	psychological treatment											0,261					0,318						0,195										0,488	0	4	0,0%	0	228.275.647	0,0%
stay at home orders for the general population (lock-down)	anxiety	0,000	0,003			0,416	0,007				0,000		0,082	0,539		0,018	0,000			0,072	0,727				0,007	0,000		0,949	0,352	0,000			0,256	9	8	52,9%	258.630.248	100.409.891	72,0%
	dejection	0,086	0,413			0,014	0,010				0,073		0,086			0,869	0,035			0,280	0,470				0,043	0,878		0,814		0,627			0,034	5	10	33,3%	177.241.433	169.933.319	51,1%
	depression	0,657	0,007			0,837	0,039				0,323		0,496	0,030		0,046	0,000			0,038	0,458				0,077	0,000		0,695	0,187	0,074			0,005	8	9	47,1%	177.741.695	181.298.444	49,5%
	depressed mood																0,152																0,222	0	2	0,0%	0	127.701.351	0,0%
	exhaustion	0,847	0,924			0,145	0,067				0,736		0,051	0,271		0,308	0,418			0,770	0,469				0,632	0,178		0,192	0,064	0,208			0,002	1	16	5,9%	68.059.863	290.980.276	19,0%
	weariness	0,051	0,135			0,728	0,004				0,054		0,596	0,052		0,716	0,043			0,029	0,705				0,005	0,060		0,247	0,623	0,337			0,007	5	12	29,4%	179.147.518	179.892.621	49,9%
	listlessness	0,163	0,608				0,850				0,002		0,442	0,228		0,474	0,739			0,752	0,277				0,104	0,001			0,597	0,000			0,397	3	12	20,0%	124.948.739	227.745.522	35,4%
	loss of appetite	0,703	0,206				0,449							0,371		0,674	0,804								0,669	0,875				0,076			0,002	1	9	10,0%	68.059.863	201.079.558	25,3%
	loss of libido	0,286	0,557								0,123					0,503																	0,729	0	5	0,0%	0	160.768.023	0,0%
	panic attack	0,646	0,024				0,753				0,003			0,664		0,000	0,075			0,813	0,167				0,986	0,472			0,033	0,333			0,009	5	9	35,7%	153.962.820	188.012.876	45,0%
	sadness	0,044	0,000			0,741	0,177				0,000		0,908	0,814		0,024	0,000			0,303	0,917				0,360	0,324		0,775	0,261	0,000			0,001	7	10	41,2%	267.742.125	91.298.014	74,6%
	bad mood	0,375	0,584								0,344					0,463	0,723								0,103	0,555				0,906			0,387	0	9	0,0%	0	315.996.172	0,0%
	insomnia	0,126	0,756			0,877	0,672				0,001		0,152	0,611		0,007	0,009			0,016	0,997				0,397	0,316		0,004	0,743	0,002			0,004	7	10	41,2%	255.570.584	103.469.555	71,2%
	sleeping problem	0,310	0,697			0,951	0,146				0,001		0,905	0,055		0,699	0,011				0,689				0,360	0,177			0,947	0,002			0,332	3	12	20,0%	174.294.318	176.493.858	49,7%
	worthlessness	0,132	0,063								0,017			0,670		0,120	0,000								0,938	0,738				0,005			0,007	4	6	40,0%	242.354.181	83.411.517	74,4%
	guilt	0,299	0,236				0,804				0,001		0,646	0,886		0,225	0,866								0,021	0,237							0,222	2	9	18,2%	105.278.354	194.567.234	35,1%
	psychology	0,110	0,006			0,015	0,181				0,482		0,003	0,071		0,079	0,661			0,430	0,533				0,016	0,177		0,824	0,022	0,266			0,820	5	12	29,4%	63.183.009	295.857.130	17,6%
	psychiatry	0,119	0,004			0,154	0,463				0,075		0,780	0,005		0,698	0,000			0,254	0,327				0,158	0,919		0,083	0,320	0,773			0,225	3	14	17,6%	80.933.454	278.106.685	22,5%
	psychologist	0,534	0,000			0,244	0,000				0,081		0,985			0,301	0,000			0,567	0,111				0,003	0,000		0,837	0,419	0,714			0,936	5	11	31,3%	130.111.914	219.158.699	37,3%
	psychotherapist	0,742	0,565				0,294				0,888			0,187		0,707	0,000			0,107	0,762				0,005	0,761		0,632	0,837	0,400			0,387	2	13	13,3%	97.599.626	249.833.943	28,1%
	psychotherapy	0,844	0,000			0,020	0,111				0,377		0,080	0,539		0,990	0,009			0,388	0,371				0,016	0,233		0,363	0,565	0,002			0,057	5	12	29,4%	157.342.685	201.697.454	43,8%
	psychological treatment	0,217									0,507						0,349													0,662			0,107	0	5	0,0%	0	251.255.245	0,0%
closure of educational institutions: daycare	anxiety	0,001	0,118	0,867	0,110	0,198	0,004	0,423			0,000	0,000	0,000	0,189		0,505	0,000		0,375	0,645	0,126	0,195	0,004	0,002	0,000	0,000	0,002	0,827	0,002	0,000			0,818	13	13	50,0%	380.228.508	121.468.066	75,8%
	dejection	0,842	0,197	0,034		0,003	0,023	0,003			0,090	0,671	0,000			0,082	0,001			0,384	0,900	0,441	0,016	0,006	0,012	0,402	0,034	0,201		0,361			0,001	11	11	50,0%	242.838.246	242.896.029	50,0%
	depression	0,743	0,013	0,453	0,132	0,971	0,019	0,152			0,458	0,000	0,912	0,008		0,012	0,003		0,244	0,012	0,729	0,063	0,000	0,000	0,002	0,557	0,255	0,006	0,985	0,000			0,000	13	13	50,0%	364.136.287	137.560.287	72,6%
	depressed mood											0,113					0,593																0,732	0	3	0,0%	0	210.868.062	0,0%
	exhaustion	0,748	0,599	0,725	0,011	0,273	0,173	0,244			0,237	0,004	0,730	0,366		0,127	0,245			0,791	0,246	0,663	0,551	0,913	0,427	0,300	0,559	0,487	0,081	0,120			0,073	2	23	8,0%	87.224.876	414.432.951	17,4%
	weariness	0,142	0,211	0,232	0,027	0,388	0,347	0,098			0,688	0,000	0,108	0,023		0,618	0,004		0,211	0,366	0,611	0,096	0,000	0,391	0,000	0,107	0,108	0,060	0,186	0,006			0,084	7	19	26,9%	259.334.227	242.362.347	51,7%
	listlessness	0,032	0,456	0,349	0,132		0,913	0,964			0,001	0,018	0,309	0,399		0,679	0,782			0,170	0,482		0,104	0,233	0,001	0,000	0,382		0,834	0,000			0,522	6	16	27,3%	254.974.652	239.822.733	51,5%
	loss of appetite	0,488	0,978	0,694	0,539		0,958					0,140		0,029		0,269	0,840						0,188		0,791	0,652				0,000			0,545	2	12	14,3%	57.102.140	323.621.224	15,0%
	loss of libido	0,208	0,166								0,029	0,185				0,228							0,718										0,125	1	6	14,3%	67.320.216	194.022.103	25,8%
	panic attack	0,416	0,336	0,372	0,631		0,814	0,841			0,016	0,382		0,185		0,324	0,012			0,001	0,606	0,053	0,200	0,507	0,800	0,025	0,158		0,007	0,113			0,016	6	16	27,3%	210.207.427	274.385.957	43,4%
	sadness	0,163	0,120	0,006	0,563	0,929	0,029	0,761			0,000	0,090	0,012	0,469		0,021	0,000			0,002	0,585	0,642	0,001	0,206	0,010	0,028	0,000	0,304	0,397	0,000			0,000	13	12	52,0%	363.467.167	138.190.660	72,5%
	bad mood	0,490	0,083								0,009	0,365				0,855	0,721						0,669	0,155	0,308	0,229	0,000			0,922			0,723	2	11	15,4%	86.649.054	354.617.832	19,6%
	insomnia	0,076	0,710	0,223	0,228	0,799	0,506	0,568			0,020	0,376	0,000	0,239		0,007	0,006			0,958	0,933	0,524	0,232	0,635	0,424	0,077	0,879	0,457	0,181	0,033			0,057	5	20	20,0%	189.977.323	311.680.504	37,9%
	sleeping problem	0,122	0,952	0,229	0,216	0,543	0,556				0,020	0,347	0,832	0,014		0,331	0,001				0,984	0,337	0,281	0,474	0,891	0,003	0,496		0,491	0,030			0,545	5	17	22,7%	194.359.753	293.223.348	39,9%
	worthlessness	0,026	0,565					0,586			0,034	0,006		0,779		0,250	0,000						0,153	0,427	0,130	0,966				0,000			0,000	6	8	42,9%	334.421.956	103.108.381	76,4%
	guilt	0,615	0,613	0,460			0,038	0,207			0,000	0,936	0,403	0,958		0,065	0,283					0,012	0,926	1,000	0,000	0,237							0,664	4	13	23,5%	116.486.857	302.589.416	27,8%
	psychology	0,152	0,348	0,158	0,230	0,920	0,042	0,272			0,375	0,001	0,000	0,126		0,444	0,853		0,141	0,066	0,161	0,043	0,910	0,004	0,000	0,613	0,034	0,075	0,852	0,000			0,044	9	17	34,6%	283.140.812	218.555.762	56,4%
	psychiatry	0,004	0,002	0,300	0,979	0,002	0,839	0,136			0,004	0,001	0,001	0,311		0,121	0,003			0,647	0,898	0,852	0,004	0,006	0,046	0,001	0,051	0,035	0,558	0,061			0,007	13	12	52,0%	386.705.437	114.952.390	77,1%
	psychologist	0,000	0,165	0,031	0,002	0,345	0,002	0,001			0,034	0,000	0,015			0,005	0,015			0,002	0,611	0,990	0,120	0,001	0,000	0,324	0,000	0,323	0,051	0,030			0,136	15	9	62,5%	375.020.093	116.868.208	76,2%
	psychotherapist	0,009	0,128	0,006			0,952	0,182			0,764	0,000		0,470		0,077	0,058			0,008	0,132	0,853	0,013	0,387	0,007	0,303	0,001	0,048	0,408	0,887			0,104	8	14	36,4%	181.965.781	304.027.311	37,4%
	psychotherapy	0,008	0,071	0,028	0,174	0,851	0,047	0,292			0,849	0,043	0,102	0,323		0,046	0,019			0,025	0,095	0,172	0,007	0,053	0,000	0,051	0,505	0,069	0,115	0,236			0,004	10	15	40,0%	300.538.800	201.119.027	59,9%
	psychological treatment	0,857									0,976	0,307					0,370						0,109							0,535			0,120	0	7	0,0%	0	351.829.541	0,0%

by Istvan-Szilard Szilagyi, Eva Eggeling, Helmar Bornemann-Cimenti, Torsten Ullrich

Measure	Effect	Country (incl. population size): Austria 8.901.064	Belgium 11.522.440	Bulgaria 6.951.482	Croatia 4.058.165	Cyprus 888.005	Czechia 10.693.939	Denmark 5.822.763	Estonia 1.328.976	Finland 5.525.292	France 67.320.216	Germany 83.166.711	Greece 10.718.565	Hungary 9.769.526	Iceland 364.134	Ireland 4.964.440	Italy 59.641.488	Latvia 1.907.675	Liechtenstein 38.747	Lithuania 2.794.090	Luxembourg 626.108	Malta 514.564	Netherlands 17.407.585	Norway 5.367.580	Poland 37.958.138	Portugal 10.295.909	Romania 19.328.838	Slovakia 5.457.873	Slovenia 2.095.861	Spain 47.332.614	Sweden 10.327.589	Switzerland 8.606.033	United Kingdom 68.059.863	significant changes	non-significant changes	percentage of countries with significant influence	significant changes weighted by population	non-significant changes weighted by population	percentage of countries with significant influence weighted by population
closure of educational institutions: primary schools	anxiety	0,000	0,002	0,938	0,141	0,761	0,022	0,242	0,047	0,557	0,042	0,002	0,000	0,097	0,232	0,039	0,212	0,040	0,098	0,041	0,126	0,195	0,004	0,002	0,000	0,000	0,000	0,820	0,001	0,000		0,000	0,818	18	13	58,1%	351.710.674	167.718.010	67,7%
	dejection	0,647	0,227	0,309		0,043	0,000	0,000	0,450	0,941	0,022	0,986	0,000		0,825	0,204	0,000	0,568		0,461	0,900	0,441	0,016	0,006	0,174	0,961	0,001	0,394		0,361		0,297	0,001	10	17	37,0%	265.248.842	238.217.543	52,7%
	depression	0,672	0,005	0,249	0,040	0,520	0,006	0,726	0,036	0,019	0,024	0,000	0,912	0,006	0,150	0,008	0,170	0,366	0,462	0,000	0,729	0,063	0,000	0,000	0,788	0,289	0,299	0,007	0,861	0,000		0,619	0,000	15	16	48,4%	344.769.310	174.659.374	66,4%
	depressed mood											0,301					0,212																0,732	0	3	0,0%	0	210.868.062	0,0%
	exhaustion	0,972	0,705	0,754	0,029	0,254	0,074	0,192		0,070	0,135	0,001	0,730	0,255	0,193	0,234	0,343			0,158	0,246	0,663	0,551	0,913	0,842	0,474	0,856	0,585	0,165	0,120		0,661	0,073	2	26	7,1%	87.224.876	428.928.410	16,9%
	weariness	0,293	0,113	0,606	0,039	0,929	0,887	0,182	0,953	0,024	0,035	0,000	0,108	0,013	0,666	0,490	0,002	0,157	0,543	0,042	0,611	0,096	0,000	0,391	0,309	0,139	0,358	0,048	0,078	0,006		0,077	0,084	10	21	32,3%	302.473.560	216.955.124	58,2%
	listlessness	0,666	0,740	0,654	0,177		0,347	0,438	0,003	0,257	0,027	0,017	0,309	0,459		0,156	0,620			0,314	0,482		0,104	0,233	0,000	0,005	0,979		0,703	0,000		0,236	0,522	6	19	24,0%	247.402.564	262.855.122	48,5%
	loss of appetite	0,674	0,348	0,105	0,490		0,142			0,403		0,247		0,014		0,023	0,030						0,188		0,004	0,208				0,000		0,057	0,545	5	11	31,3%	159.666.206	235.188.483	40,4%
	loss of libido	0,387	0,535								0,058	0,078				0,538							0,718									0,339	0,125	0	8	0,0%	0	269.948.352	0,0%
	panic attack	0,354	0,088	0,169	0,278		0,786	0,505	0,608	0,543	0,973	0,765		0,101	0,572	0,037	0,009	0,023		0,213	0,606	0,053	0,200	0,507	0,373	0,001	0,020		0,036	0,113		0,070	0,016	7	20	25,9%	166.294.074	336.031.420	33,1%
	sadness	0,112	0,001	0,172	0,460	0,202	0,296	0,826	0,175	0,156	0,567	0,153	0,012	0,325	0,427	0,123	0,472	0,711		0,000	0,585	0,642	0,001	0,206	0,015	0,387	0,000	0,406	0,200	0,000		0,022	0,000	9	21	30,0%	223.728.166	295.661.771	43,1%
	bad mood	0,263	0,249								0,108	0,310				0,635	0,754						0,669	0,155	0,596	0,117	0,000			0,922		0,812	0,723	1	13	7,1%	19.328.838	430.544.081	4,3%
	insomnia	0,556	0,340	0,691	0,332	0,941	0,000	0,360	0,697	0,050	0,209	0,693	0,000	0,383	0,539	0,079	0,022	0,337		0,156	0,933	0,524	0,232	0,635	0,017	0,001	0,488	0,139	0,237	0,033		0,812	0,057	7	23	23,3%	182.165.945	337.223.992	35,1%
	sleeping problem	0,284	0,807	0,612	0,202	0,699	0,027		0,258	0,383	0,194	0,955	0,832	0,014		0,097	0,029	0,252			0,984	0,337	0,281	0,474	0,000	0,001	0,775		0,453	0,030		0,193	0,545	6	20	23,1%	175.691.614	329.259.463	34,8%
	worthlessness	0,054	0,141					0,632	0,489	0,003	0,817	0,010		0,916		0,181	0,620						0,153	0,427	0,050	0,744				0,000		0,117	0,000	5	12	29,4%	242.042.618	210.948.020	53,4%
	guilt	0,181	0,616	0,252			0,000	0,487		0,498	0,012	0,830	0,403	0,760		0,474	0,465					0,012	0,926	1,000	0,326	0,025						0,297	0,664	4	15	21,1%	88.824.628	344.382.970	20,5%
	psychology	0,551	0,002	0,177	0,238	0,224	0,466	0,055	0,018	0,062	0,952	0,000	0,000	0,062	0,691	0,034	0,909	0,028	0,598	0,001	0,161	0,043	0,910	0,004	0,000	0,493	0,000	0,110	0,734	0,000		0,189	0,044	13	18	41,9%	294.964.494	224.464.190	56,8%
	psychiatry	0,201	0,019	0,090	0,986	0,107	0,192	0,165	0,162	0,240	0,191	0,005	0,001	0,176		0,058	0,248	0,149		0,116	0,898	0,852	0,004	0,006	0,021	0,021	0,015	0,100	0,850	0,061		0,001	0,007	10	19	34,5%	272.431.662	246.594.141	52,5%
	psychologist	0,426	0,002	0,058	0,001	0,452	0,012	0,136	0,002	0,975	0,031	0,000	0,015		0,768	0,055	0,935	0,436		0,004	0,611	0,990	0,120	0,001	0,015	0,000	0,000	0,941	0,149	0,030		0,505	0,136	13	16	44,8%	311.886.181	197.734.230	61,2%
	psychotherapist	0,456	0,641	0,002			0,022	0,559	0,216	0,812	0,028	0,000		0,354	0,768	0,086	0,324	0,004		0,240	0,132	0,853	0,013	0,387	0,077	0,684	0,013	0,128	0,441	0,887		0,424	0,104	7	20	25,9%	206.776.446	296.948.756	41,0%
	psychotherapy	0,894	0,000	0,000	0,245	0,068	0,461	0,869	0,069	0,124	0,044	0,007	0,102	0,194		0,020	0,045	0,483		0,895	0,095	0,172	0,007	0,053	0,680	0,196	0,936	0,069	0,284	0,236		0,394	0,004	8	21	27,6%	319.034.225	199.991.578	61,5%
	psychological treatment	0,309									0,724	0,222					0,133						0,109							0,535			0,120	0	7	0,0%	0	351.829.541	0,0%
closure of educational institutions: secondary schools	anxiety	0,000	0,004	0,615	0,461	0,525	0,562	0,722	0,059	0,992	0,000	0,002	0,000	0,778	0,248	0,019	0,660	0,920	0,098	0,001	0,098	0,195	0,028	0,006	0,000	0,000	0,000	0,974	0,001	0,000	0,139	0,000	0,496	15	17	46,9%	337.780.084	191.976.189	63,8%
	dejection	0,153	0,266	0,257		0,122	0,000	0,000	0,356	0,552	0,721	0,986	0,000		0,308	0,173	0,000	0,749		0,746	0,577	0,441	0,005	0,002	0,000	0,827	0,001	0,830		0,361	0,000	0,297	0,000	10	18	35,7%	245.326.348	268.467.626	47,7%
	depression	0,344	0,001	0,156	0,409	0,402	0,012	0,097	0,027	0,000	0,947	0,000	0,111	0,002	0,297	0,003	0,034	0,033	0,462	0,000	0,292	0,063	0,000	0,009	0,251	0,432	0,207	0,002	0,638	0,000	0,006	0,619	0,000	16	16	50,0%	345.267.681	184.488.592	65,2%
	depressed mood											0,301					0,041																0,447	1	2	33,3%	59.641.488	151.226.574	28,3%
	exhaustion	0,938	0,408	0,545	0,574	0,237	0,050	0,225		0,014	0,233	0,001	0,826	0,494	0,229	0,244	0,026			0,882	0,302	0,663	0,960	0,869	0,869	0,253	0,962	0,429	0,118	0,120	0,113	0,661	0,239	4	25	13,8%	159.027.430	367.453.445	30,2%
	weariness	0,039	0,107	0,860	0,108	0,290	0,637	0,441	0,637	0,070	0,693	0,000	0,727	0,570	0,153	0,652	0,499	0,539	0,543	0,011	0,949	0,096	0,004	0,325	0,226	0,064	0,479	0,202	0,165	0,006	0,000	0,077	0,260	6	26	18,8%	169.929.653	359.826.620	32,1%
	listlessness	0,381	0,440	0,896	0,239		0,502	0,398	0,009	0,837	0,001	0,017	0,537	0,622		0,091	0,842			0,862	0,242		0,011	0,258	0,000	0,000	0,877		0,987	0,000	0,016	0,236	0,416	8	18	30,8%	275.137.738	245.447.537	52,9%
	loss of appetite	0,770	0,256	0,251	0,390		0,921			0,377		0,247		0,254		0,015	0,037						0,857		0,000	0,801				0,000	0,368	0,057	0,252	4	13	23,5%	149.896.680	255.285.598	37,0%
	loss of libido	0,234	0,764								0,010	0,078				0,662							0,345									0,339	0,069	1	7	12,5%	67.320.216	202.628.136	24,9%
	panic attack	0,226	0,028	0,010	0,700		0,244	0,876	0,591	0,226	0,025	0,765		0,011	0,390	0,046	0,001	0,553		0,200	0,297	0,053	0,033	0,920	0,303	0,071	0,010		0,002	0,113	0,026	0,070	0,017	11	17	39,3%	277.389.328	235.263.755	54,1%
	sadness	0,024	0,002	0,027	0,740	0,023	0,084	0,312	0,110	0,677	0,000	0,153	0,002	0,028	0,819	0,088	0,923	0,399		0,002	0,775	0,642	0,000	0,231	0,000	0,079	0,000	0,486	0,334	0,000	0,088	0,022	0,000	14	17	45,2%	317.558.459	212.159.067	59,9%
	bad mood	0,724	0,433								0,010	0,310				0,565	0,843						0,233	0,261	0,967	0,066	0,000			0,922	0,216	0,812	0,842	2	13	13,3%	86.649.054	373.551.454	18,8%
	insomnia	0,065	0,467	0,522	0,093	0,628	0,001	0,630	0,926	0,006	0,004	0,693	0,083	0,524	0,078	0,105	0,149	0,009		0,024	0,801	0,524	0,238	0,291	0,003	0,061	0,503	0,034	0,104	0,033	0,768	0,812	0,088	8	23	25,8%	178.989.837	350.727.689	33,8%
	sleeping problem	0,248	0,838	0,643	0,087	0,977	0,142		0,337	0,067	0,003	0,955	0,748	0,173		0,178	0,001	0,012			0,646	0,337	0,117	0,594	0,000	0,007	0,997		0,427	0,030	0,838	0,193	0,467	6	21	22,2%	224.456.040	290.822.626	43,6%
	worthlessness	0,207	0,204					0,251	0,455	0,018	0,161	0,010		0,827		0,208	0,457						0,346	0,291	0,002	0,795				0,000	0,003	0,117	0,000	6	12	33,3%	252.370.207	210.948.020	54,5%
	guilt	0,297	0,730	0,121			0,000	0,156		0,083	0,000	0,830	0,172	0,619		0,322	0,359					0,012	0,845	0,549	0,059	0,192					0,107	0,297	0,309	3	17	15,0%	78.528.719	365.006.468	17,7%
	psychology	0,078	0,002	0,039	0,616	0,543	0,311	0,004	0,005	0,929	0,761	0,000	0,000	0,065	0,759	0,048	0,071	0,043	0,598	0,001	0,561	0,043	0,657	0,011	0,000	0,823	0,000	0,017	0,750	0,000	0,926	0,189	0,024	16	16	50,0%	313.196.612	216.559.661	59,1%
	psychiatry	0,058	0,004	0,001	0,227	0,174	0,047	0,105	0,403	0,141	0,048	0,005	0,001	0,511		0,063	0,001	0,978		0,325	0,866	0,852	0,002	0,135	0,979	0,007	0,014	0,028	0,605	0,061	0,217	0,001	0,008	13	17	43,3%	379.170.942	150.182.450	71,6%
	psychologist	0,784	0,001	0,002	0,087	0,818	0,279	0,080	0,002	0,030	0,019	0,000	0,001		0,150	0,034	0,833	0,309		0,007	0,242	0,990	0,238	0,003	0,791	0,059	0,000	0,517	0,080	0,030	0,278	0,505	0,282	12	18	40,0%	266.321.244	253.626.756	51,2%
	psychotherapist	0,473	0,543	0,098			0,007	0,031	0,145	0,623	0,721	0,000		0,386	0,152	0,088	0,925	0,405		0,527	0,547	0,853	0,014	0,498	0,000	0,412	0,025	0,149	0,140	0,887	0,859	0,424	0,462	6	22	21,4%	174.377.974	339.674.817	33,9%
	psychotherapy	0,588	0,000	0,003	0,814	0,428	0,333	0,980	0,084	0,221	0,868	0,007	0,011	0,104		0,031	0,001	0,824		0,821	0,073	0,172	0,043	0,301	0,005	0,061	0,778	0,049	0,367	0,236	0,450	0,394	0,011	10	20	33,3%	305.848.585	223.504.807	57,8%
	psychological treatment	0,080									0,750	0,222					0,143						0,305							0,535	0,087		0,122	0	8	0,0%	0	362.157.130	0,0%
closure of educational institutions: higher education	anxiety	0,422	0,125	0,656	0,461	0,696	0,096	0,434	0,384	0,992	0,000		0,000	0,319	0,248	0,124	0,125	0,194	0,098	0,002	0,126	0,000	0,038	0,213	0,000	0,000	0,002	0,467	0,004	0,000	0,829	0,000		11	19	36,7%	224.372.413	154.157.286	59,3%
	dejection	0,491	0,102	0,009		0,002	0,000	0,000	0,451	0,552	0,049		0,000		0,308	0,230	0,001	0,112		0,704	0,900	0,784	0,003	0,007	0,072	0,590	0,034	0,227		0,361	0,000	0,002		12	14	46,2%	223.074.083	139.493.317	61,5%
	depression	0,147	0,000	0,274	0,409	0,685	0,033	0,083	0,043	0,000	0,000		0,023	0,000	0,297	0,008	0,026	0,002	0,462	0,000	0,729	0,017	0,000	0,159	0,001	0,196	0,255	0,005	0,924	0,000	0,000	0,324		17	13	56,7%	305.185.010	73.344.689	80,6%
	depressed mood																0,098																	0	1	0,0%	0	59.641.488	0,0%
	exhaustion	0,218	0,942	0,993	0,574	0,089	0,045	0,273		0,014	0,149		0,543	0,582	0,229	0,133	0,008			0,756	0,246	0,164	0,973	0,299	0,453	0,192	0,559	0,642	0,049	0,120	0,079	0,288		4	23	14,8%	77.956.580	297.297.721	20,8%
	weariness	0,281	0,354	0,192	0,108	0,264	0,932	0,567	0,995	0,070	0,036		0,607	0,506	0,153	0,637	0,517	0,288	0,543	0,025	0,611	0,001	0,094	0,180	0,000	0,057	0,108	0,110	0,230	0,006	0,000	0,048		7	23	23,3%	174.853.244	203.676.455	46,2%
	listlessness	0,330	0,022	0,167	0,239		0,280	0,521	0,002	0,837	0,061		0,578	0,750		0,306	0,475			0,438	0,482		0,099	0,734	0,000	0,000	0,382		0,677	0,000	0,000	0,252		6	18	25,0%	118.765.666	250.593.035	32,2%
	loss of appetite	0,246	0,911	0,157	0,390		0,172			0,377				0,248		0,052	0,464						0,388		0,575	0,858				0,000	0,432	0,432		1	14	6,7%	47.332.614	206.623.090	18,6%
	loss of libido	0,609	0,989								0,114					0,746							0,727									0,045		1	5	16,7%	8.606.033	110.115.745	7,2%
	panic attack	0,050	0,010	0,828	0,700		0,211	0,303	0,610	0,226	0,036			0,198	0,390	0,053	0,004	0,052		0,244	0,606	0,166	0,003	0,882	0,939	0,132	0,158		0,002	0,113	0,095	0,000		7	19	26,9%	175.494.687	185.931.822	48,6%
	sadness	0,019	0,000	0,152	0,740	0,505	0,108	0,074	0,044	0,677	0,000		0,002	0,039	0,819	0,354	0,002	0,630		0,000	0,585	0,195	0,000	0,030	0,001	0,054	0,000	0,318	0,475	0,000	0,000	0,008		15	14	51,7%	318.324.742	60.166.210	84,1%
	bad mood	0,961	0,393								0,174					0,657	0,230						0,290	0,190	0,444	0,085	0,000			0,922	0,138	0,664		1	12	7,7%	19.328.838	289.645.096	6,3%
	insomnia	0,648	0,436	0,723	0,093	0,631	0,000	0,854	0,559	0,006	0,013		0,008	0,283	0,078	0,126	0,006	0,002		0,025	0,933	0,262	0,810	0,599	0,227	0,052	0,879	0,944	0,161	0,033	0,015	0,292		9	20	31,0%	216.261.468	162.229.484	57,1%
	sleeping problem	0,047	0,962	0,232	0,087	0,914	0,154		0,053	0,067	0,012		0,438	0,069		0,146	0,000	0,011			0,984	0,096	0,124	0,067	0,524	0,004	0,496		0,506	0,030	0,928	0,221		6	19	24,0%	195.398.966	168.653.126	53,7%
	worthlessness	0,049	0,635					0,273	0,489	0,018	0,044			0,959		0,136	0,001						0,110	0,136	0,049	0,984				0,000	0,006	0,064		7	9	43,8%	237.006.401	75.085.252	75,9%
	guilt	0,529	0,734	0,081			0,000	0,886		0,083	0,218		0,772	0,002		0,436	0,084					0,000	0,588	0,955	0,000	0,167					0,782	0,209		4	14	22,2%	58.936.167	233.372.446	20,2%
	psychology	0,310	0,097	0,179	0,616	0,023	0,203	0,000	0,049	0,929	0,906		0,000	0,030	0,759	0,082	0,112	0,000	0,598	0,002	0,161	0,126	0,660	0,056	0,000	0,970	0,034	0,074	0,682	0,000	0,381	0,217		10	20	33,3%	137.849.190	240.680.509	36,4%
	psychiatry	0,006	0,000	0,215	0,227	0,048	0,125	0,575	0,245	0,141	0,577		0,000	0,185		0,010	0,000	0,070		0,177	0,898	0,638	0,009	0,146	0,056	0,021	0,051	0,014	0,470	0,061	0,285	0,233		9	19	32,1%	129.797.369	248.329.449	34,3%
	psychologist	0,160	0,342	0,230	0,087	0,323	0,071	0,035	0,005	0,030	0,040		0,000		0,150	0,003	0,101	0,419		0,005	0,611	0,063	0,642	0,392	0,000	0,022	0,000	0,875	0,046	0,030	0,001	0,009		14	14	50,0%	234.419.324	134.302.102	63,6%
	psychotherapist	0,014	0,212	0,056			0,007	0,000	0,118	0,623	0,005			0,990	0,152	0,048	0,140	0,135		0,337	0,132	0,317	0,171	0,148	0,022	0,859	0,001	0,026	0,290	0,887	0,533	0,004		9	17	34,6%	169.053.304	193.772.913	46,6%
	psychotherapy	0,153	0,000	0,089	0,814	0,029	0,949	0,786	0,271	0,221	0,598		0,000	0,760		0,027	0,002	0,952		0,522	0,095	0,004	0,084	0,709	0,000	0,191	0,505	0,110	0,308	0,236	0,637	0,000		8	20	28,6%	134.813.673	243.313.145	35,7%
	psychological treatment	0,154									0,777						0,503						0,199							0,535	0,099			0	6	0,0%	0	210.930.556	0,0%

Measure	Effect	Austria (8.901.064)	Belgium (11.522.440)	Bulgaria (6.951.482)	Croatia (4.058.165)	Cyprus (888.005)	Czechia (10.693.939)	Denmark (5.822.763)	Estonia (1.328.976)	Finland (5.525.292)	France (67.320.216)	Germany (83.166.711)	Greece (10.718.565)	Hungary (9.769.526)	Iceland (364.134)	Ireland (4.964.440)	Italy (59.641.488)	Latvia (1.907.675)	Liechtenstein (38.747)	Lithuania (2.794.090)	Luxembourg (626.108)	Malta (514.564)	Netherlands (17.407.585)	Norway (5.367.580)	Poland (37.958.138)	Portugal (10.295.909)	Romania (19.328.838)	Slovakia (5.457.873)	Slovenia (2.095.861)	Spain (47.332.614)	Sweden (10.327.589)	Switzerland (8.606.033)	United Kingdom (68.059.863)	significant changes	non-significant changes	percentage of countries with significant influence	significant changes weighted by population	non-significant changes weighted by population	percentage of countries with significant influence weighted by population
interventions are in place to limit all mass public gatherings	anxiety	0,001	0,000	0,225	0,038	0,155	0,678	0,910			0,000	0,000	0,005	0,615		0,000	0,069	0,969	0,671	0,013		0,000	0,198	0,005	0,016	0,000		0,704	0,000	0,000	0,356	0,001	0,003	16	11	59,3%	373.676.253	128.906.672	74,4%
	dejection	0,223	0,083	0,004		0,001	0,000	0,000			0,134	0,430	0,000			0,584	0,000	0,201		0,400		0,501	0,012	0,002	0,000	0,557		0,144		0,017	0,001	0,009	0,686	12	11	52,2%	221.715.781	264.904.845	45,6%
	depression	0,788	0,042	0,572	0,010	0,972	0,723	0,879			0,000	0,917	0,066	0,002		0,005	0,368	0,000	0,085	0,004		0,008	0,000	0,003	0,000	0,000		0,001	0,029	0,000	0,183	0,294	0,044	16	11	59,3%	296.826.539	205.756.386	59,1%
	depressed mood											0,485					0,299																0,646	0	3	0,0%	0	210.868.062	0,0%
	exhaustion	0,924	0,113	0,668	0,280	0,237	0,290	0,252			0,526	0,000	0,908	0,468		0,443	0,000			0,577		0,133	0,296	0,699	0,282	0,124		0,228	0,056	0,000	0,490	0,272	0,006	4	21	16,0%	258.200.676	242.435.827	51,6%
	weariness	0,483	0,959	0,094	0,040	0,469	0,059	0,642			0,002	0,637	0,385	0,381		0,147	0,569	0,654	0,525	0,005		0,030	0,773	0,589	0,000	0,033		0,032	0,487	0,000	0,010	0,216	0,015	10	17	37,0%	254.119.021	248.463.904	50,6%
	listlessness	0,558	0,027	0,133	0,197		0,214	0,576			0,001	0,001	0,266	0,484		0,587	0,901			0,947			0,665	0,307	0,000	0,008			0,065	0,000	0,034	0,642	0,920	7	15	31,8%	267.923.617	225.852.444	54,3%
	loss of appetite	0,653	0,345	0,358	0,923		0,147					0,253		0,263		0,758	0,093						0,967		0,000	0,121				0,009	0,003	0,581	0,000	4	12	25,0%	163.678.204	235.978.782	41,0%
	loss of libido	0,267	0,050								0,098	0,000				0,810							0,995									0,083	0,229	2	6	25,0%	94.689.151	175.259.201	35,1%
	panic attack	0,014	0,873	0,780	0,434		0,048	0,368			0,007	0,000		0,005		0,000	0,000	0,514		0,030		0,243	0,001	0,949	0,266	0,070			0,109	0,003	0,049	0,000	0,018	13	10	56,5%	398.985.158	86.494.577	82,2%
	sadness	0,253	0,000	0,157	0,100	0,500	0,798	0,037			0,000	0,146	0,003	0,045		0,002	0,137	0,177		0,015		0,383	0,000	0,062	0,101	0,385		0,475	0,808	0,342	0,376	0,021	0,001	10	16	38,5%	206.985.521	295.558.657	41,2%
	bad mood	0,560	0,064								0,140	0,656				0,130	0,645						0,574	0,204	0,637	0,026				0,081	0,252	0,276	0,576	1	13	7,1%	10.295.909	430.575.761	2,3%
	insomnia	0,078	0,009	0,883	0,635	0,521	0,000	0,617			0,355	0,620	0,304	0,654		0,002	0,079	0,003		0,050		0,126	0,529	0,461	0,000	0,003		0,951	0,047	0,025	0,384	0,725	0,406	9	17	34,6%	129.565.106	372.979.072	25,8%
	sleeping problem	0,718	0,003	0,165	0,947	0,352	0,459				0,350	0,818	0,970	0,207		0,849	0,018	0,027				0,052	0,531	0,644	0,000	0,009			0,294	0,021	0,266	0,694	0,054	6	17	26,1%	168.658.264	319.811.188	34,5%
	worthlessness	0,501	0,009					0,885			0,047	0,000		0,801		0,216	0,004						0,286	0,430	0,022	0,403				0,000	0,244	0,325	0,210	6	10	37,5%	306.941.607	149.522.352	67,2%
	guilt	0,366	0,847	0,031			0,000	0,814			0,000	0,063	0,902	0,898		0,889	0,104					0,002	0,630	0,561	0,446	0,245					0,113	0,092	0,232	4	15	21,1%	85.480.201	352.529.694	19,5%
	psychology	0,084	0,115	0,040	0,232	0,817	0,698	0,000			0,404	0,000	0,001	0,105		0,413	0,053	0,009	0,255	0,017		0,880	0,936	0,003	0,003	0,071		0,200	0,937	0,000	0,229	0,677	0,000	10	17	37,0%	270.079.481	232.503.444	53,7%
	psychiatry	0,052	0,003	0,026	0,959	0,003	0,052	0,759			0,073	0,000	0,012	0,605		0,388	0,000	0,301		0,546		0,925	0,008	0,133	0,412	0,915		0,042	0,043	0,000	0,156	0,744	0,859	10	16	38,5%	245.182.624	257.361.554	48,8%
	psychologist	0,000	0,000	0,036	0,002	0,358	0,976	0,147			0,000	0,909	0,001			0,217	0,006	0,617		0,006		0,113	0,942	0,003	0,000	0,000		0,304	0,011	0,000	0,676	0,009	0,000	15	10	60,0%	351.623.508	141.151.144	71,4%
	psychotherapist	0,034	0,000	0,009			0,001	0,000			0,000	0,006		0,572		0,958	0,063	0,730		0,743		0,488	0,240	0,203	0,000	0,023		0,127	0,457	0,487	0,920	0,021	0,247	10	13	43,5%	251.238.695	235.640.748	51,6%
	psychotherapy	0,000	0,747	0,030	0,755	0,580	0,281	0,640			0,004	0,000	0,001	0,071		0,977	0,358	0,493		0,794		0,309	0,014	0,130	0,000	0,000		0,054	0,725	0,379	0,859	0,076	0,676	8	18	30,8%	242.719.670	259.824.508	48,3%
	psychological treatment	0,082									0,505	0,028					0,349						0,074							0,002	0,268		0,411	2	6	25,0%	130.499.325	231.657.805	36,0%
closure of entertainment venues	anxiety	0,000	0,000	0,490	0,050	0,776	0,009	0,832	0,194	0,638	0,000	0,000	0,000	0,716	0,133	0,001	0,035	0,781	0,629	0,076	0,010	0,171	0,021	0,012	0,000	0,000	0,034	0,458	0,001	0,000		0,097	0,055	17	14	54,8%	401.399.661	118.029.023	77,3%
	dejection	0,153	0,168	0,006		0,035	0,000	0,000	0,932	0,470	0,251	0,648	0,000		0,102	0,169	0,001	0,012		0,879	0,634	0,281	0,004	0,006	0,005	0,684	0,639	0,952		0,146		0,029	0,003	12	15	44,4%	234.023.116	269.443.269	46,5%
	depression	0,344	0,904	0,363	0,427	0,600	0,093	0,039	0,035	0,003	0,000	0,017	0,021	0,000	0,832	0,000	0,012	0,000	0,060	0,040	0,993	0,233	0,000	0,020	0,782	0,270	0,220	0,062	0,074	0,000		0,073	0,000	15	16	48,4%	391.127.384	128.301.300	75,3%
	depressed mood											0,012					0,187																0,772	1	2	33,3%	83.166.711	127.701.351	39,4%
	exhaustion	0,938	0,084	0,667	0,023	0,192	0,047	0,430		0,028	0,626	0,456	0,724	0,307	0,133	0,090	0,000			0,522	0,183	0,534	0,217	0,175	0,873	0,153	0,424	0,619	0,066	0,000		0,465	0,097	5	23	17,9%	127.251.498	388.901.788	24,7%
	weariness	0,039	0,609	0,100	0,008	0,483	0,890	0,842	0,641	0,077	0,159	0,000	0,432	0,319	0,411	0,907	0,392	0,682	0,663	0,014	0,178	0,390	0,247	0,010	0,776	0,034	0,197	0,337	0,037	0,000		0,012	0,121	9	22	29,0%	172.618.027	346.810.657	33,2%
	listlessness	0,381	0,135	0,296	0,019		0,225	0,829	0,003	0,224	0,000	0,001	0,440	0,614		0,874	0,730			0,768	0,873		0,546	0,050	0,000	0,000	0,429		0,638	0,000		0,845	0,296	8	17	32,0%	256.828.309	253.429.377	50,3%
	loss of appetite	0,770	0,305	0,569	0,460		0,093			0,281		0,083		0,157		0,154	0,073						0,796		0,001	0,614				0,007		0,379	0,001	3	13	18,8%	153.350.615	241.504.074	38,8%
	loss of libido	0,234	0,169								0,011	0,002				0,737							0,995									0,859	0,143	2	6	25,0%	150.486.927	119.461.425	55,7%
	panic attack	0,226	0,116	0,691	0,930		0,464	0,713	0,505	0,461	0,001	0,000		0,068	0,378	0,000	0,000	0,288		0,013	0,492	0,034	0,001	0,325	0,284	0,061	0,018		0,002	0,000		0,025	0,000	12	15	44,4%	381.232.303	121.093.191	75,9%
	sadness	0,024	0,000	0,294	0,400	0,034	0,225	0,269	0,175	0,218	0,000	0,737	0,002	0,062	0,608	0,007	0,032	0,270		0,014	0,844	0,334	0,000	0,291	0,002	0,012	0,001	0,384	0,595	0,850		0,450	0,000	13	17	43,3%	319.800.641	199.589.296	61,6%
	bad mood	0,724	0,010								0,931	0,163				0,191	0,873						0,552	0,051	0,472	0,047	0,001			0,046		0,959	0,811	4	10	28,6%	88.479.801	361.393.118	19,7%
	insomnia	0,065	0,002	0,849	0,591	0,402	0,000	0,514	0,373	0,008	0,002	0,090	0,001	0,164	0,193	0,005	0,124	0,002		0,045	0,423	0,144	0,372	0,485	0,009	0,121	0,578	0,000	0,623	0,014		0,009	0,019	13	17	43,3%	282.861.178	236.528.759	54,5%
	sleeping problem	0,248	0,003	0,109	0,201	0,880	0,185		0,966	0,757	0,002	0,065	0,543	0,040		0,425	0,120	0,024			0,190	0,241	0,152	0,003	0,000	0,040	0,213		0,543	0,012		0,202	0,203	8	18	30,8%	191.474.098	313.476.979	37,9%
	worthlessness	0,207	0,002					0,124	0,386	0,040	0,000	0,000		0,785		0,132	0,002						0,213	0,302	0,028	0,754				0,000		0,596	0,001	8	9	47,1%	380.526.762	72.463.876	84,0%
	guilt	0,297	0,687	0,058			0,000	0,254		0,648	0,000	0,119	0,434	0,003		0,998	0,481					0,009	0,957	0,257	0,154	0,145						0,172	0,181	4	15	21,1%	88.298.245	344.909.353	20,4%
	psychology	0,078	0,419	0,060	0,289	0,391	0,585	0,000	0,081	0,014	0,395	0,004	0,000	0,004	0,104	0,970	0,087	0,006	0,196	0,283	0,266	0,225	0,861	0,004	0,000	0,989	0,001	0,060	0,821	0,000		0,779	0,116	10	21	32,3%	226.897.702	292.530.982	43,7%
	psychiatry	0,058	0,258	0,039	0,757	0,047	0,252	0,954	0,393	0,325	0,738	0,001	0,000	0,222		0,287	0,000	0,153		0,872	0,903	0,705	0,006	0,003	0,175	0,007	0,005	0,001	0,115	0,000		0,363	0,009	12	17	41,4%	334.616.513	184.409.290	64,5%
	psychologist	0,784	0,029	0,080	0,002	0,961	0,018	0,684	0,009	0,842	0,001	0,378	0,000		0,004	0,852	0,182	0,118		0,032	0,332	0,474	0,792	0,072	0,040	0,028	0,000	0,626	0,264	0,000		0,070	0,786	12	17	41,4%	223.716.024	285.904.387	43,9%
	psychotherapist	0,473	0,001	0,023			0,008	0,000	0,216	0,779	0,000	0,012		0,692	0,004	0,524	0,724	0,735		0,381	0,740	0,360	0,119	0,199	0,003	0,522	0,003	0,487	0,597	0,650		0,232	0,888	9	18	33,3%	243.128.661	260.596.541	48,3%
	psychotherapy	0,588	0,030	0,084	0,122	0,211	0,481	0,354	0,639	0,272	0,008	0,250	0,000	0,424		0,450	0,311	0,601		0,763	0,054	0,472	0,018	0,265	0,252	0,097	0,452	0,793	0,945	0,425		0,049	0,005	6	23	20,7%	183.634.702	335.391.101	35,4%
	psychological treatment	0,080									0,750	0,009					0,729						0,069							0,003			0,163	2	5	28,6%	130.499.325	221.330.216	37,1%
closure of public transport	anxiety				0,149																								0,284					0	2	0,0%	0	6.154.026	0,0%
	dejection																																	0	0		0	0	
	depression				0,031																								0,467					1	1	50,0%	4.058.165	2.095.861	65,9%
	depressed mood																																	0	0		0	0	
	exhaustion				0,074																								0,067					0	2	0,0%	0	6.154.026	0,0%
	weariness				0,054																								0,108					0	2	0,0%	0	6.154.026	0,0%
	listlessness				0,302																								0,320					0	2	0,0%	0	6.154.026	0,0%
	loss of appetite				0,854																													0	1	0,0%	0	4.058.165	0,0%
	loss of libido																																	0	0		0	0	
	panic attack				0,549																								0,071					0	2	0,0%	0	6.154.026	0,0%
	sadness				0,411																								0,424					0	2	0,0%	0	6.154.026	0,0%
	bad mood																																	0	0		0	0	
	insomnia				0,374																								0,607					0	2	0,0%	0	6.154.026	0,0%
	sleeping problem				0,454																								0,449					0	2	0,0%	0	6.154.026	0,0%
	worthlessness																																	0	0		0	0	
	guilt																																	0	0		0	0	
	psychology				0,164																								0,451					0	2	0,0%	0	6.154.026	0,0%
	psychiatry				0,496																								0,247					0	2	0,0%	0	6.154.026	0,0%
	psychologist				0,000																								0,643					1	1	50,0%	4.058.165	2.095.861	65,9%
	psychotherapist																												0,958					0	1	0,0%	0	2.095.861	0,0%
	psychotherapy				0,201																								0,640					0	2	0,0%	0	6.154.026	0,0%
	psychological treatment																																	0	0		0	0	

Measure	Effect	Austria (8.901.064)	Belgium (11.522.440)	Bulgaria (6.951.482)	Croatia (4.058.165)	Cyprus (888.005)	Czechia (10.693.939)	Denmark (5.822.763)	Estonia (1.328.976)	Finland (5.525.292)	France (67.320.216)	Germany (83.166.711)	Greece (10.718.565)	Hungary (9.769.526)	Iceland (364.134)	Ireland (4.964.440)	Italy (59.641.488)	Latvia (1.907.675)	Liechtenstein (38.747)	Lithuania (2.794.090)	Luxembourg (626.108)	Malta (514.564)	Netherlands (17.407.585)	Norway (5.367.580)	Poland (37.958.138)	Portugal (10.295.909)	Romania (19.328.838)	Slovakia (5.457.873)	Slovenia (2.095.861)	Spain (47.332.614)	Sweden (10.327.589)	Switzerland (8.606.033)	United Kingdom (68.059.863)	significant changes	non-significant changes	percentage of countries with significant influence	significant changes weighted by population	non-significant changes weighted by population	percentage of countries with significant influence weighted by population
closure of gyms sports centers	anxiety	0,001	0,006	0,490	0,265	0,754	0,177	0,499	0,045	0,465	0,000	0,000	0,000	0,537	0,488	0,000	0,006	0,510	0,688	0,001	0,049	0,094	0,019	0,023	0,000	0,000	0,009	0,454	0,224	0,000		0,000	0,197	17	14	54,8%	397.280.795	122.147.889	76,5%
	dejection	0,223	0,179	0,006		0,038	0,000	0,000	0,660	0,519	0,175	0,970	0,000		0,038	0,468	0,000	0,007		0,222	0,885	0,357	0,006	0,009	0,570	0,774	0,083	0,956		0,163		0,003	0,006	12	15	44,4%	196.429.112	307.037.273	39,0%
	depression	0,788	0,005	0,363	0,052	0,548	0,106	0,168	0,052	0,001	0,000	0,747	0,140	0,011	0,642	0,001	0,055	0,000	0,001	0,004	0,223	0,141	0,000	0,013	0,022	0,388	0,185	0,028	0,194	0,001		0,259	0,000	14	17	45,2%	285.426.079	234.002.605	55,0%
	depressed mood											0,056					0,398																0,353	0	3	0,0%	0	210.868.062	0,0%
	exhaustion	0,924	0,565	0,667	0,005	0,167	0,077	0,250		0,015	0,486	0,263	0,763	0,922	0,432	0,512	0,000			0,807	0,672	0,908	0,538	0,627	0,642	0,184	0,359	0,737	0,122	0,557		0,286	0,001	4	24	14,3%	137.284.808	378.868.478	26,6%
	weariness	0,483	0,050	0,100	0,000	0,949	0,808	0,750	0,660	0,050	0,045	0,015	0,117	0,463	0,031	0,011	0,717	0,723	1,000	0,076	0,516	0,095	0,756	0,156	0,040	0,080	0,256	0,519	0,301	0,017		0,004	0,003	11	20	35,5%	338.878.046	180.550.638	65,2%
	listlessness	0,558	0,165	0,296	0,013		0,308	0,963	0,001	0,393	0,000	0,004	0,775	0,925		0,220	0,929			0,917	0,289		0,205	0,600	0,000	0,001	0,365		0,409	0,000		0,845	0,817	7	18	28,0%	251.460.729	258.796.957	49,3%
	loss of appetite	0,653	0,206	0,569	0,209		0,231			0,443		0,008		0,995		0,580	0,016						0,547		0,073	0,974				0,010		0,882	0,003	4	12	25,0%	258.200.676	136.654.013	65,4%
	loss of libido	0,267	0,557								0,031	0,000				0,755							0,614									0,105	0,632	2	6	25,0%	150.486.927	119.461.425	55,7%
	panic attack	0,014	0,011	0,691	0,408		0,116	0,610	0,650	0,516	0,000	0,000		0,026	0,505	0,000	0,000	0,163		0,613	0,683	0,005	0,005	0,546	0,626	0,009	0,034		0,039	0,629		0,000	0,001	14	13	51,9%	371.594.538	130.730.956	74,0%
	sadness	0,253	0,001	0,294	0,269	0,515	0,299	0,122	0,205	0,302	0,000	0,144	0,000	0,049	0,738	0,025	0,505	0,266		0,335	0,843	0,720	0,000	0,126	0,006	0,053	0,000	0,306	0,307	0,000		0,034	0,000	11	19	36,7%	302.988.258	216.401.679	58,3%
	bad mood	0,560	0,777								0,712	0,070				0,090	0,901						0,936	0,179	0,514	0,056	0,000			0,532		0,249	0,393	1	13	7,1%	19.328.838	430.544.081	4,3%
	insomnia	0,078	0,879	0,849	0,741	0,563	0,000	0,754	0,568	0,004	0,017	0,028	0,000	0,448	0,037	0,002	0,378	0,007		0,004	0,294	0,191	0,668	0,482	0,458	0,320	0,909	0,000	0,592	0,001		0,185	0,012	12	18	40,0%	308.305.412	211.084.525	59,4%
	sleeping problem	0,718	0,817	0,109	0,531	0,898	0,188		0,862	0,730	0,015	0,037	0,801	0,750		0,880	0,111	0,075			0,436	0,109	0,333	0,919	0,132	0,234	0,547		0,859	0,001		0,491	0,147	3	23	11,5%	197.819.541	307.131.536	39,2%
	worthlessness	0,501	0,241					0,047	0,518	0,098	0,000	0,008		0,689		0,158	0,001						0,473	0,171	0,118	0,408				0,000		0,273	0,006	6	11	35,3%	331.343.655	121.646.983	73,1%
	guilt	0,366	0,652	0,058			0,000	0,198		0,385	0,000	0,245	0,749	0,061		0,886	0,528					0,000	0,653	0,281	0,000	0,192						0,026	0,306	5	14	26,3%	125.092.890	308.114.708	28,9%
	psychology	0,084	0,004	0,060	0,620	0,027	0,178	0,000	0,075	0,014	0,307	0,192	0,000	0,551	0,128	0,904	0,010	0,006	0,890	0,075	0,193	0,085	0,799	0,121	0,000	0,673	0,003	0,033	0,043	0,005		0,150	0,831	12	19	38,7%	208.199.552	311.229.132	40,1%
	psychiatry	0,052	0,020	0,039	0,394	0,170	0,263	0,845	0,202	0,313	0,542	0,030	0,000	0,075		0,161	0,000	0,038		0,153	0,884	0,343	0,024	0,063	0,105	0,004	0,034	0,001	0,261	0,425		0,316	0,039	11	18	37,9%	294.458.429	224.567.374	56,7%
	psychologist	0,000	0,000	0,080	0,097	0,048	0,048	0,293	0,013	0,949	0,000	0,136	0,000		0,054	0,025	0,002	0,090		0,202	0,518	0,596	0,520	0,050	0,000	0,018	0,000	0,503	0,538	0,422		0,003	0,647	14	15	48,3%	257.535.631	252.084.780	50,5%
	psychotherapist	0,034	0,250	0,023			0,022	0,000	0,319	0,540	0,000	0,609		0,713	0,055	0,787	0,077	0,684		0,070	0,719	0,195	0,247	0,585	0,831	0,134	0,003	0,403	0,926	0,737		0,001	0,160	7	20	25,9%	127.624.335	376.100.867	25,3%
	psychotherapy	0,000	0,000	0,084	0,039	0,036	0,950	0,344	0,345	0,424	0,003	0,007	0,000	0,399		0,976	0,416	0,643		0,211	0,146	0,487	0,033	0,366	0,117	0,056	0,323	0,712	0,313	0,296		0,001	0,008	10	19	34,5%	280.648.647	238.377.156	54,1%
	psychological treatment	0,082									0,457	0,021					0,177						0,133							0,248			0,032	2	5	28,6%	151.226.574	200.602.967	43,0%
closure of non-essential shops	anxiety	0,000	0,000	0,673	0,149	0,473	0,241	0,635	0,081		0,000	0,000	0,002	0,358		0,000	0,048		0,884	0,208	0,727	0,022	0,088		0,000	0,000	0,034	0,989	0,180			0,022	0,256	12	14	46,2%	322.938.406	135.992.983	70,4%
	dejection	0,143	0,249	0,041		0,119	0,000	0,000	0,805		0,088	0,266	0,000			0,711	0,102			0,499	0,470	0,419	0,004		0,050	0,774	0,639	0,309				0,013	0,034	8	14	36,4%	166.218.368	276.750.722	37,5%
	depression	0,530	0,112	0,402	0,031	0,809	0,077	0,737	0,069		0,696	0,103	0,108	0,021		0,006	0,006		0,883	0,073	0,458	0,107	0,203		0,553	0,388	0,220	0,024	0,833			0,213	0,005	6	20	23,1%	151.951.355	306.980.034	33,1%
	depressed mood											0,029					0,004																0,222	2	1	66,7%	142.808.199	68.059.863	67,7%
	exhaustion	0,955	0,838	0,444	0,074	0,427	0,152	0,320			0,960	0,602	0,651	0,215		0,445	0,289			0,463	0,469	0,859	0,620		0,718	0,184	0,424	0,443	0,127			0,906	0,002	1	23	4,2%	68.059.863	389.503.803	14,9%
	weariness	0,160	0,023	0,331	0,054	0,818	0,680	0,657	0,417		0,258	0,003	0,967	0,020		0,097	0,538		0,732	0,016	0,705	0,091	0,241		0,443	0,080	0,197	0,429	0,144			0,913	0,007	5	21	19,2%	175.312.630	283.618.759	38,2%
	listlessness	0,229	0,817	0,722	0,302		0,447	0,293	0,000		0,018	0,035	0,460	0,326		0,553	0,937			0,709	0,277		0,002		0,000	0,001	0,429		0,651			0,511	0,397	6	16	27,3%	217.477.535	234.554.665	48,1%
	loss of appetite	0,814	0,246	0,751	0,854		0,624					0,675		0,133		0,572	0,748						0,934		0,001	0,974						0,824	0,002	2	12	14,3%	106.018.001	235.978.782	31,0%
	loss of libido	0,478	0,228								0,025	0,022				0,935							0,560									0,408	0,729	2	6	25,0%	150.486.927	119.461.425	55,7%
	panic attack	0,200	0,046	0,105	0,549		0,262	0,585	0,480		0,004	0,000		0,516		0,000	0,003			0,026	0,167	0,019	0,058		0,182	0,009	0,018		0,184			0,037	0,009	11	11	50,0%	336.214.592	105.613.607	76,1%
	sadness	0,111	0,000	0,018	0,411	0,475	0,080	0,100	0,301		0,000	0,703	0,013	0,483		0,003	0,015			0,007	0,917	0,885	0,001		0,012	0,053	0,001	0,312	0,148			0,073	0,001	11	14	44,0%	306.667.145	152.225.497	66,8%
	bad mood	0,891	0,993								0,467	0,151				0,178	0,531						0,606		0,485	0,056	0,001					0,872	0,387	1	11	8,3%	19.328.838	377.843.887	4,9%
	insomnia	0,141	0,659	0,152	0,374	0,167	0,001	0,386	0,624		0,002	0,029	0,185	0,206		0,004	0,113			0,065	0,997	0,082	0,033		0,000	0,320	0,578	0,549	0,865			0,598	0,004	7	18	28,0%	289.570.892	169.321.750	63,1%
	sleeping problem	0,708	0,183	0,419	0,454	0,815	0,249		0,806		0,002	0,048	0,540	0,039		0,970	0,032				0,689	0,162	0,906		0,000	0,234	0,213		0,404			0,296	0,332	5	17	22,7%	257.856.079	186.961.837	58,0%
	worthlessness	0,393	0,143					0,974	0,533		0,107	0,003		0,681		0,241	0,005						0,764		0,037	0,408						0,386	0,007	4	10	28,6%	248.826.200	145.938.952	63,0%
	guilt	0,376	0,737	0,464			0,000	0,342			0,001	0,064	0,586	0,531		0,719	0,071					0,002	0,754		0,555	0,192						0,149	0,222	3	14	17,6%	78.528.719	343.786.007	18,6%
	psychology	0,413	0,011	0,536	0,164	0,924	0,245	0,012	0,115		0,689	0,080	0,000	0,096		0,491	0,050		0,290	0,150	0,533	0,186	0,101		0,000	0,673	0,001	0,550	0,830			0,587	0,820	6	20	23,1%	144.992.232	313.939.157	31,6%
	psychiatry	0,398	0,467	0,353	0,496	0,004	0,135	0,737	0,119		0,040	0,139	0,042	0,035		0,442	0,000			0,572	0,327	0,498	0,208		0,129	0,004	0,005	0,095	0,023			0,108	0,225	8	17	32,0%	180.058.408	278.834.234	39,2%
	psychologist	0,976	0,015	0,061	0,000	0,358	0,068	0,417	0,002		0,012	0,211	0,108			0,193	0,005			0,003	0,111	0,382	0,218		0,096	0,018	0,000	0,027	0,095			0,233	0,936	9	15	37,5%	181.747.995	267.375.121	40,5%
	psychotherapist	0,865	0,789	0,066			0,029	0,002	0,123		0,531	0,051		0,173		0,835	0,016			0,112	0,762	0,299	0,467		0,016	0,134	0,003	0,107	0,882			0,106	0,387	5	17	22,7%	133.445.166	309.782.741	30,1%
	psychotherapy	0,834	0,000	0,188	0,201	0,524	0,921	0,666	0,570		0,421	0,498	0,607	0,306		0,977	0,008			0,811	0,371	0,827	0,852		0,476	0,056	0,452	0,921	0,305			0,530	0,057	2	23	8,0%	71.163.928	387.728.714	15,5%
	psychological treatment	0,048									0,399	0,492					0,550						0,634										0,107	1	5	16,7%	8.901.064	295.595.863	2,9%
closure of places of worship	anxiety	0,000	0,002			0,507	0,091	0,972	0,284				0,005			0,000	0,000	0,512		0,645		0,000	0,126			0,002	0,034		0,085				0,680	8	9	47,1%	125.887.308	110.998.757	53,1%
	dejection	0,393	0,060			0,258	0,012	0,000	0,804				0,034			0,976	0,000	0,005		0,384		0,812	0,351			0,520	0,639						0,001	6	10	37,5%	156.844.293	77.945.911	66,8%
	depression	0,615	0,000			0,892	0,012	0,006	0,112				0,799			0,027	0,011	0,058		0,012		0,160	0,000			0,022	0,220		0,517				0,000	9	8	52,9%	191.202.517	45.683.548	80,7%
	depressed mood																0,563																0,824	0	2	0,0%	0	127.701.351	0,0%
	exhaustion	0,969	0,920			0,463	0,018	0,555					0,598			0,939	0,200			0,791		0,326	0,711			0,236	0,424		0,400				0,027	2	13	13,3%	78.753.802	154.895.612	33,7%
	weariness	0,458	0,019			0,489	0,028	0,976	0,941				0,104			0,168	0,003	1,000		0,366		0,440	0,004			0,467	0,197		0,585				0,063	4	13	23,5%	99.265.452	137.620.613	41,9%
	listlessness	0,291	0,559				0,648	0,779	0,194				0,813			0,868	0,981			0,170			0,662			0,025	0,429		0,049				0,676	2	12	14,3%	12.391.770	221.184.051	5,3%
	loss of appetite	0,826	0,320				0,556									0,903	0,997						0,904			0,324							0,449	0	8	0,0%	0	191.486.728	0,0%
	loss of libido	0,264	0,650													0,509							0,470										0,197	0	5	0,0%	0	110.855.392	0,0%
	panic attack	0,183	0,010				0,849	0,643	0,899							0,000	0,008	0,331		0,001		0,221	0,152			0,174	0,018		0,272				0,005	6	9	40,0%	166.311.159	58.968.336	73,8%
	sadness	0,059	0,000			0,678	0,419	0,988	0,351				0,893			0,140	0,000	0,778		0,002		0,295	0,045			0,015	0,001		0,177				0,000	7	10	41,2%	189.050.213	47.835.852	79,8%
	bad mood	0,200	0,360													0,250	0,761						0,776			0,193	0,001						0,498	1	7	12,5%	19.328.838	180.792.789	9,7%
	insomnia	0,181	0,899			0,377	0,285	0,192	0,043				0,070			0,080	0,004	0,378		0,958		0,066	0,652			0,211	0,578		0,281				0,020	3	14	17,6%	129.030.327	107.855.738	54,5%
	sleeping problem	0,458	0,889			0,977	0,173		0,400				0,830			0,897	0,003	0,610				0,361	0,188			0,333	0,213		0,940				0,532	1	14	6,7%	59.641.488	168.627.724	26,1%
	worthlessness	0,357	0,200					0,021	0,642							0,505	0,001						0,449			0,354							0,000	3	6	33,3%	133.524.114	54.420.414	71,0%
	guilt	0,712	0,496				0,289	0,169					0,358			0,120	0,290					0,015	0,515			0,315							0,783	1	10	9,1%	514.564	208.028.056	0,2%
	psychology	0,652	0,004			0,551	0,438	0,102	0,845				0,001			0,444	0,939	0,462		0,066		0,564	0,109			0,085	0,001		0,953				0,088	3	14	17,6%	41.569.843	195.316.222	17,5%
	psychiatry	0,236	0,000			0,012	0,143	0,057	0,034				0,621			0,980	0,012	0,314		0,647		0,506	0,002			0,000	0,005		0,735				0,020	8	9	47,1%	188.473.104	48.412.961	79,6%
	psychologist	0,629	0,000			0,503	0,000	0,000	0,099				0,830			0,001	0,039	0,020		0,002		0,203	0,003			0,493	0,000		0,140				0,260	9	8	52,9%	134.083.258	102.802.807	56,6%
	psychotherapist	0,959	0,059				0,939	0,826	0,417							0,385	0,148	0,962		0,008		0,261	0,000			0,038	0,003		0,295				0,098	4	11	26,7%	49.826.422	175.453.073	22,1%
	psychotherapy	0,783	0,000			0,694	0,384	0,772	0,791				0,136			0,222	0,050	0,826		0,025		0,112	0,000			0,001	0,452		0,137				0,003	6	11	35,3%	169.721.375	67.164.690	71,6%
	psychological treatment	0,092															0,254						0,051										0,128	0	4	0,0%	0	154.010.000	0,0%

**Measure**	**Effect**	**Country** (incl. population size): Austria 8.901.064	Belgium 11.522.440	Bulgaria 6.951.482	Croatia 4.058.165	Cyprus 888.005	Czechia 10.693.939	Denmark 5.822.763	Estonia 1.328.976	Finland 5.525.292	France 67.320.216	Germany 83.166.711	Greece 10.718.565	Hungary 9.769.526	Iceland 364.134	Ireland 4.964.440	Italy 59.641.488	Latvia 1.907.675	Liechtenstein 38.747	Lithuania 2.794.090	Luxembourg 626.108	Malta 514.564	Netherlands 17.407.585	Norway 5.367.580	Poland 37.958.138	Portugal 10.295.909	Romania 19.328.838	Slovakia 5.457.873	Slovenia 2.095.861	Spain 47.332.614	Sweden 10.327.589	Switzerland 8.606.033	United Kingdom 68.059.863	significant changes	non-significant changes	percentage of countries with significant influence	significant changes weighted by population	non-significant changes weighted by population	percentage of countries with significant influence weighted by population
closure of restaurants and cafes	anxiety	0,000	0,000	0,311	0,251	0,874	0,094	0,971	0,409	0,638	0,000	0,000	0,000	0,755	0,698	0,000	0,264	0,995	0,747	0,001	0,018	0,082	0,035	0,021	0,000	0,000	0,009	0,813	0,821	0,000		0,000	0,055	15	16	48,4%	336.310.331	183.118.353	64,7%
	dejection	0,375	0,453	0,099		0,026	0,000	0,000	0,910	0,470	0,251	0,124	0,000		0,025	0,331	0,155	0,734		0,453	0,930	0,419	0,017	0,013	0,000	0,683	0,083	0,346		0,627		0,008	0,003	10	17	37,0%	165.886.605	337.579.780	32,9%
	depression	0,761	0,575	0,533	0,099	0,832	0,070	0,082	0,110	0,003	0,000	0,704	0,137	0,013	0,181	0,000	0,403	0,004	0,048	0,000	0,595	0,110	0,000	0,032	0,174	0,149	0,185	0,749	0,329	0,074		0,266	0,000	10	21	32,3%	183.155.014	336.273.670	35,3%
	depressed mood											0,232					0,062																0,772	0	3	0,0%	0	210.868.062	0,0%
	exhaustion	0,919	0,093	0,725	0,006	0,136	0,088	0,373		0,028	0,626	0,147	0,694	0,488	0,986	0,353	0,108			0,911	0,139	0,919	0,186	0,683	0,831	0,021	0,359	0,332	0,225	0,208		0,938	0,097	3	25	10,7%	19.879.366	496.273.920	3,9%
	weariness	0,827	0,410	0,435	0,000	0,458	0,707	0,854	0,991	0,077	0,159	0,035	0,361	0,471	0,088	0,025	0,222	0,713	0,688	0,042	0,660	0,212	0,063	0,223	0,165	0,036	0,256	0,335	0,281	0,337		0,102	0,121	5	26	16,1%	105.279.315	414.149.369	20,3%
	listlessness	0,792	0,131	0,265	0,003		0,327	0,668	0,009	0,224	0,000	0,001	0,885	0,575		0,306	0,810			0,619	0,537		0,142	0,550	0,000	0,000	0,365		0,597	0,000		0,531	0,296	7	18	28,0%	251.460.729	258.796.957	49,3%
	loss of appetite	0,938	0,203	0,528	0,471		0,238			0,281		0,285		0,329		0,409	0,491						0,841		0,000	0,519				0,076		0,976	0,001	2	14	12,5%	106.018.001	288.836.688	26,8%
	loss of libido	0,112	0,343								0,011	0,000				0,556							0,995									0,117	0,143	2	6	25,0%	150.486.927	119.461.425	55,7%
	panic attack	0,012	0,103	0,054	0,442		0,198	0,996	0,413	0,461	0,001	0,000		0,053	0,256	0,000	0,059	0,202		0,314	0,224	0,007	0,000	0,964	0,234	0,205	0,034		0,145	0,333		0,000	0,000	9	18	33,3%	278.269.314	224.056.180	55,4%
	sadness	0,082	0,000	0,001	0,120	0,337	0,208	0,405	0,205	0,218	0,000	0,970	0,002	0,023	0,440	0,034	0,573	0,243		0,024	0,624	0,651	0,000	0,281	0,001	0,054	0,000	0,988	0,349	0,000		0,021	0,000	13	17	43,3%	312.733.830	206.656.107	60,2%
	bad mood	0,930	0,026								0,931	0,385				0,058	0,517						0,428	0,091	0,868	0,087	0,000			0,906		0,381	0,811	2	12	14,3%	30.851.278	419.021.641	6,9%
	insomnia	0,025	0,013	0,020	0,851	0,404	0,000	0,804	0,332	0,008	0,002	0,216	0,001	0,783	0,233	0,004	0,369	0,043		0,008	0,181	0,126	0,536	0,193	0,001	0,026	0,909	0,000	0,328	0,002		0,144	0,019	15	15	50,0%	300.403.600	218.986.337	57,8%
	sleeping problem	0,245	0,011	0,711	0,796	0,677	0,208		0,517	0,757	0,002	0,100	0,483	0,444		0,720	0,097	0,076			0,279	0,162	0,337	0,900	0,000	0,030	0,547		0,821	0,002		0,810	0,203	5	21	19,2%	174.429.317	330.521.760	34,5%
	worthlessness	0,794	0,001					0,048	0,664	0,040	0,000	0,000		0,624		0,210	0,843						0,126	0,306	0,003	0,496				0,005		0,267	0,001	8	9	47,1%	326.708.037	126.282.601	72,1%
	guilt	0,245	0,762	0,936			0,000	0,047		0,648	0,000	0,044	0,843	0,594		0,866	0,676					0,002	0,538	0,481	0,094	0,077						0,025	0,181	6	13	31,6%	176.124.226	257.083.372	40,7%
	psychology	0,023	0,250	0,977	0,472	0,054	0,204	0,000	0,046	0,014	0,395	0,000	0,000	0,184	0,645	0,954	0,095	0,024	0,054	0,015	0,085	0,233	0,620	0,011	0,000	0,989	0,003	0,176	0,126	0,266		0,725	0,116	11	20	35,5%	182.819.692	336.608.992	35,2%
	psychiatry	0,772	0,367	0,742	0,331	0,060	0,235	0,748	0,770	0,325	0,738	0,000	0,000	0,759		0,097	0,226	0,425		0,262	0,354	0,498	0,022	0,055	0,992	0,001	0,034	0,044	0,982	0,773		0,836	0,009	7	22	24,1%	214.435.344	304.590.459	41,3%
	psychologist	0,001	0,044	0,568	0,319	0,625	0,034	0,310	0,165	0,842	0,001	0,856	0,000		0,185	0,051	0,707	0,706		0,011	0,066	0,420	0,517	0,001	0,630	0,005	0,000	0,661	0,565	0,714		0,012	0,786	10	19	34,5%	155.548.674	354.071.737	30,5%
	psychotherapist	0,355	0,001	0,073			0,021	0,003	0,570	0,779	0,000	0,010		0,638	0,188	0,817	0,214	0,936		0,592	0,032	0,299	0,129	0,294	0,000	0,705	0,003	0,939	0,767	0,400		0,021	0,888	9	18	33,3%	245.045.186	258.680.016	48,6%
	psychotherapy	0,000	0,005	0,162	0,013	0,140	0,970	0,795	0,283	0,272	0,008	0,008	0,000	0,077		0,825	0,684	0,904		0,852	0,717	0,827	0,014	0,222	0,005	0,511	0,323	0,164	0,332	0,002		0,036	0,005	11	18	37,9%	365.051.394	153.974.409	70,3%
	psychological treatment	0,060									0,750	0,014					0,298						0,036							0,662			0,163	2	5	28,6%	100.574.296	251.255.245	28,6%
teleworking recommandations	anxiety	0,001	0,000	0,935	0,050		0,647	0,906		0,930	0,000	0,000	0,000			0,000	0,001	0,075		0,711	0,004	0,000	0,464	0,005	0,000	0,000	0,000	0,888	0,000	0,001	0,360	0,000	0,995	17	10	63,0%	382.418.734	134.948.151	73,9%
	dejection	0,371	0,522	0,445			0,000	0,000		0,670	0,320	0,851	0,000			0,017	0,000	0,208		0,218	0,860	0,472	0,026	0,000	0,000	0,439	0,000	0,700		0,970	0,000	0,204	0,000	11	14	44,0%	250.290.788	260.922.071	49,0%
	depression	0,293	0,236	0,331	0,427		0,102	0,019		0,000	0,000	0,002	0,720			0,000	0,149	0,009		0,000	0,765	0,011	0,000	0,017	0,095	0,089	0,049	0,879	0,000	0,289	0,000	0,104	0,000	14	13	51,9%	294.603.067	222.763.818	56,9%
	depressed mood											0,341					0,774																0,660	0	3	0,0%	0	210.868.062	0,0%
	exhaustion	0,143	0,102	0,092	0,023		0,291	0,319		0,020	0,789	0,000	0,836			0,046	0,000			0,235	0,667	0,038	0,857	0,067	0,928	0,104	0,008	0,311	0,003	0,202	0,100	0,360	0,012	9	17	34,6%	247.355.222	268.103.988	48,0%
	weariness	0,315	0,242	0,083	0,008		0,056	0,825		0,042	0,009	0,000	0,031			0,070	0,216	0,126		0,050	0,489	0,000	0,680	0,001	0,282	0,081	0,792	0,202	0,027	0,573	0,000	0,095	0,005	11	16	40,7%	259.948.496	257.418.389	50,2%
	listlessness	0,394	0,122	0,449	0,019		0,167	0,753		0,902	0,000	0,100	0,621			0,231	0,407			0,766	0,058		0,305	0,002	0,000	0,000	0,757		0,945	0,004	0,000	0,001	0,982	8	16	33,3%	191.266.244	318.220.529	37,5%
	loss of appetite	0,500	0,106	0,707	0,460		0,135			0,620		0,624				0,124	0,005						0,304		0,014	0,739				0,081	0,330	0,919	0,045	3	13	18,8%	165.659.489	229.753.263	41,9%
	loss of libido	0,224	0,545								0,025	0,000				0,854							0,453									0,042	0,423	3	5	37,5%	159.092.960	110.855.392	58,9%
	panic attack	0,000	0,111	0,029	0,930		0,070	0,650		0,344	0,000	0,000				0,000	0,000	0,002		0,000	0,167	0,782	0,000	0,737	0,269	0,218	0,076		0,023	0,604	0,085	0,000	0,002	12	13	48,0%	331.816.508	169.373.939	66,2%
	sadness	0,078	0,000	0,317	0,400		0,334	0,324		0,754	0,000	0,050	0,017			0,738	0,282	0,668		0,000	0,781	0,819	0,000	0,004	0,976	0,033	0,748	0,465	0,894	0,002	0,000	0,409	0,002	11	16	40,7%	334.313.162	183.053.723	64,6%
	bad mood	0,981	0,205								0,658	0,937				0,087	0,816						0,639	0,039	0,412	0,177	0,904			0,244	0,138	0,318	0,144	1	14	6,7%	5.367.580	454.832.928	1,2%
	insomnia	0,958	0,321	0,543	0,591		0,000	0,590		0,008	0,021	0,274	0,000			0,194	0,397	0,006		0,492	0,969	0,324	0,403	0,968	0,904	0,054	0,440	0,001	0,550	0,140	0,050	0,814	0,163	7	20	25,9%	111.951.149	405.415.736	21,6%
	sleeping problem	0,234	0,057	0,570	0,201		0,121			0,072	0,021	0,075	0,860			0,244	0,041	0,045			0,842	0,005	0,096	0,798	0,008	0,041	0,530		0,756	0,138	0,608	0,504	0,491	6	18	25,0%	177.637.990	325.654.169	35,3%
	worthlessness	0,064	0,006					0,065		0,033	0,005	0,042				0,048	0,014						0,023	0,128	0,188	0,746				0,106	0,007	0,762	0,001	9	7	56,3%	327.935.624	124.284.101	72,5%
	guilt	0,597	0,911	0,414			0,000	0,165		0,163	0,000	0,096	0,831			0,218	0,252					0,001	0,848	0,680	0,010	0,118					0,610	0,937	0,652	4	15	21,1%	116.486.857	317.278.804	26,9%
	psychology	0,001	0,205	0,573	0,289		0,152	0,000		0,860	0,394	0,000	0,000			0,059	0,000	0,001		0,000	0,116	0,336	0,937	0,013	0,000	0,857	0,000	0,072	0,386	0,847	0,091	0,195	0,076	10	17	37,0%	235.606.912	281.759.973	45,5%
	psychiatry	0,365	0,355	0,667	0,757		0,001	0,833		0,156	0,647	0,084	0,000			0,001	0,000	0,024		0,441	0,803	0,526	0,007	0,003	0,050	0,022	0,573	0,004	0,686	0,988	0,076	0,634	0,047	11	16	40,7%	232.473.055	284.893.830	44,9%
	psychologist	0,000	0,000	0,317	0,002		0,205	0,480		0,044	0,000	0,124	0,000			0,161	0,000	0,524		0,010	0,042	0,013	0,451	0,168	0,019	0,002	0,021	0,640	0,795	0,705	0,003	0,010	0,020	16	11	59,3%	326.198.362	191.168.523	63,0%
	psychotherapist	0,749	0,002	0,074			0,000	0,000		0,650	0,000	0,467				0,975	0,001	0,016		0,216	0,157	0,222	0,116	0,684	0,000	0,325	0,064	0,881	0,819	0,238	0,914	0,028	0,020	9	16	36,0%	271.532.555	231.057.600	54,0%
	psychotherapy	0,000	0,004	0,317	0,122		0,229	0,477		0,265	0,025	0,000	0,000			0,043	0,925	0,555		0,429	0,351	0,025	0,001	0,139	0,000	0,354	0,815	0,291	0,040	0,009	0,588	0,012	0,002	13	14	48,1%	368.568.094	148.798.791	71,2%
	psychological treatment	0,009									0,359	0,646					0,386						0,104							0,687	0,090		0,106	1	7	12,5%	8.901.064	353.256.066	2,5%
closure of workplaces	anxiety					0,067		0,755			0,000												0,010						0,430					2	3	40,0%	84.727.801	8.806.629	90,6%
	dejection					0,320		0,000			0,512												0,008											2	2	50,0%	23.230.348	68.208.221	25,4%
	depression					0,963		0,002			0,144												0,003						0,292					2	3	40,0%	23.230.348	70.304.082	24,8%
	depressed mood																																	0	0		0	0	
	exhaustion					0,551		0,412			0,653												0,479						0,165					0	5	0,0%	0	93.534.430	0,0%
	weariness					0,937		0,886			0,333												0,010						0,357					1	4	20,0%	17.407.585	76.126.845	18,6%
	listlessness							0,747			0,008												0,011						0,494					2	2	50,0%	84.727.801	7.918.624	91,5%
	loss of appetite																						0,869											0	1	0,0%	0	17.407.585	0,0%
	loss of libido										0,218												0,203											0	2	0,0%	0	84.727.801	0,0%
	panic attack							0,710			0,012												0,019						0,084					2	2	50,0%	84.727.801	7.918.624	91,5%
	sadness					0,576		0,604			0,000												0,000						0,253					2	3	40,0%	84.727.801	8.806.629	90,6%
	bad mood										0,027												0,784											1	1	50,0%	67.320.216	17.407.585	79,5%
	insomnia					0,387		0,273			0,009												0,124						0,394					1	4	20,0%	67.320.216	26.214.214	72,0%
	sleeping problem					0,171					0,009												0,558						0,894					1	3	25,0%	67.320.216	20.391.451	76,8%
	worthlessness							0,152			0,034												0,356											1	2	33,3%	67.320.216	23.230.348	74,3%
	guilt							0,022			0,002												0,836											2	1	66,7%	73.142.979	17.407.585	80,8%
	psychology					0,454		0,333			0,615												0,517						0,056					0	5	0,0%	0	93.534.430	0,0%
	psychiatry					0,057		0,366			0,001												0,014						0,522					2	3	40,0%	84.727.801	8.806.629	90,6%
	psychologist					0,911		0,000			0,000												0,281						0,424					2	3	40,0%	73.142.979	20.391.451	78,2%
	psychotherapist							0,310			0,126												0,118						0,880					0	4	0,0%	0	92.646.425	0,0%
	psychotherapy					0,814		0,462			0,087												0,033						0,371					1	4	20,0%	17.407.585	76.126.845	18,6%
	psychological treatment										0,943												0,487											0	2	0,0%	0	84.727.801	0,0%

by Istvan-Szilard Szilagyi, Eva Eggeling, Helmar Bornemann-Cimenti, Torsten Ullrich
