# Supplementary material for: Impact of the pandemic and its containment measures in Europe upon aspects of affective impairments: a Google Trends informetrics study
Source: Psychol Med. 2023 Jun 26;53(16):7685–97. doi: 10.1017/S0033291723001563 (PMC10755220; doi:10.1017/S0033291723001563)
Supplement: Szilagyi et al. supplementary material 2 — Szilagyi et al. supplementary material [file S0033291723001563sup002.pdf]

Country	Country Code	Language	anxiety	anxious	dejection	depressed	depression	depressed	exhaustion	exhausted	guilt	insomnia
Austria	AT	de	Angst	ängstlich	Niedergeschlagenheit	niedergeschlagen	Depression	depressiv	Erschöpfung	erschöpft	Schuldgefühle	Schlaflosigkeit
Belgium	BE	nl (59%)	Angst	bang	Neerslachtigheid	omlaag	Depressie	depressief	Uitputting	uitgeput	Schuldgevoel	Slapeloosheid
Belgium	BE	fr (40%)	anxiété	anxieux	abattement	abattu	dépression	déprimé	épuisement	épuisé	culpabilité	insomnie
Belgium	BE	de (1%)	Angst	ängstlich	Niedergeschlagenheit	niedergeschlagen	Depression	depressiv	Erschöpfung	erschöpft	Schuldgefühle	Schlaflosigkeit
Bulgaria	BG	bg	тревожност	неспокоен	дежекция	в депресия	депресия	в депресия	изтощение	изчерпан	вина	безсъние
Croatia	HR	hr	strah	plašljiv	potištenost	potišten	depresija	depresivan	iscrpljenost	iscrpljen	osjećaj krivice	nesanica
Cyprus	CY	gr (81%)	Άγχος	φοβισ ένος	Κάτω	κάτω	Κατάθλιψη	καταθλιπτικός	Εξάντληση	εξαντλη ένο	Ενοχή	Αϋπνία
Cyprus	CY	tr (1%)	korku	korkak	yılgın	yılgın	çöküntü	depresif	bitkinlik	tükenmiş	suçluluk duygusu	uykusuzluk
Czechia	CZ	cz	Strach	vyděšený	sklíčenost	sklíčený	Deprese	depresivní	Vyčerpání	vyčerpaný	pocit	nespavost
Denmark	DK	dk	Frygt	bange	Lavpris	ned	Depression	depressiv	Udmattelse	udmattet	Skyld	Søvnløshed
Estonia	EE	ee	Hirm	hirmunud	Madalamalt	alla	Depressioon	depressiiv	Kurnatus	kurnatud	Süütunne	Unetus
Finland	FI	fi (87%)	Pelko	peloissaan	Lowdown	alas	Masennus	depressiivinen	Uupumus	uupunut	Syyllisyys	Unettomuus
Finland	FI	se (5%)	Rädsla	rädd	Lågt sänkt	ner	Depression	depressiv	Utmattning	utmattad	Skuldkänslor	Sömnlöshet
France	FR	fr	anxiété	anxieux	abattement	abattu	dépression	déprimé	épuisement	épuisé	culpabilité	insomnie
Germany	DE	de	Angst	ängstlich	Niedergeschlagenheit	niedergeschlagen	Depression	depressiv	Erschöpfung	erschöpft	Schuldgefühle	Schlaflosigkeit
Greece	GR	gr	Άγχος	φοβισ ένος	Κάτω	κάτω	Κατάθλιψη	καταθλιπτικός	Εξάντληση	εξαντλη ένο	Ενοχή	Αϋπνία
Hungary	HU	hu	Félelem	ijedt	rossz hangulat	rossz hangulat	Depresszió	depressziós	Kimerültség	kimerült	Bűntudat	Álmatlanság
Iceland	IS	is	kvíði	hræddur	Brottvísun	niðurdreginn	þunglyndi	þunglyndislegt	örmögnun	búinn	Sektarkennd	svefnleysi
Ireland	IE	gb (85%)	anxiety	anxious	dejection	depressed	depression	depressed	exhaustion	exhausted	guilt	insomnia

by Istvan-Szilard Szilagyi, Eva Eggeling, Helmar Bornemann-Ciments, Torsten Ullrich

Country	Country Code	Language	anxiety	anxious	dejection	depressed	depression	depressed	exhaustion	exhausted	guilt	insomnia
Ireland	IE	ie (10%)	imní	scanraithe	Díochlaonadh	dejected	dúlagar	dúlagar	ídithe	traochta	Mothúcháin chiontachta	insomnia
Italy	IT	it	paura	spaventato	abbattimento	giù	depressione	depressivo	esaurimento	esaurito	senso di colpa	insonnia
Latvia	LV	lv	Bailes	nobijies	Pazemināt	depresija	Depresija	depressiv	Izsīkums	erschöpft	Vainas sajūta	Bezmiegs
Liechtenstein	LI	de	Angst	ängstlich	Niedergeschlagenheit	niedergeschlagen	Depression	depressiv	Erschöpfung	erschöpft	Schuldgefühle	Schlaflosigkeit
Lithuania	LT	lt	Baimė	išsigandęs	Nusileidimas	žemyn	Depresija	depresija	Išsekimas	išsekęs	Kaltės jausmas	Nemiga
Luxembourg	LU	fr (56%)	anxiété	anxieux	abattement	abattu	dépression	déprimé	épuisement	épuisé	culpabilité	insomnie
Luxembourg	LU	lu (20%)	Angschtgefiller	Angscht	depriméiert	depriméiert	Depressioun	depressiv	Iwwermiddung	erschöpft	Scholdgefiller	Insomnia
Luxembourg	LU	de (6%)	Angst	ängstlich	Niedergeschlagenheit	niedergeschlagen	Depression	depressiv	Erschöpfung	erschöpft	Schuldgefühle	Schlaflosigkeit
Malta	MT	mt (86%)	ansjetà	imbeżża	Ċaħda	imdejjaq	depressjoni	depressiv	eżawriment	eżawrit	Sentimenti ta 'ħtija	nuqqas ta 'rqad
Malta	MT	gb (12%)	anxiety	anxious	dejection	depressed	depression	depressed	exhaustion	exhausted	guilt	insomnia
Netherlands	NL	nl	Angst	bang	Neerslachtigheid	omlaag	Depressie	depressief	Uitputting	uitgeput	Schuldgevoel	Slapeloosheid
Norway	NO	no	angst	engstelig	motløshet	deprimert	depresjon	deprimert	utmattelse	utslitt	skyld	søvnløshet
Poland	PL	pl	Strach	przestraszony	Niski poziom	w dó	Depresja	depresja	Wyczerpanie	wyczerpany	Wina	Bezsenność
Portugal	PT	pt	ansiedade	assustada	Desânimo	abatido	depressão	depressivo	exaustão	Exausta	Sentimentos de culpa	insônia
Romania	RO	ro	anxietate	speriat	Dejecție	abătut	depresie	depresiv	epuizare	epuizat	Sentimente de vinovăție	insomnie
Slovakia	SK	sk	úzkosť	vystrašený	Odmietnutie	skľúčený	depresia	depresívny	vyčerpanie	vyčerpaný	Pocity viny	nespavosť
Slovenia	SI	si	anksioznost	zaskrbljeni	potrtosti	potrt	depresija	depresivno	izčrpanost	izčrpan	Občutek krivde	nespečnost
Spain	ES	es	ansiedad	asustado	abatimiento	abatido	depresión	depresivo	agotamiento	exhausto	sentimientos de culpa	insomnio
Sweden	SE	se	Rädsla	rädd	Lågt sänkt	ner	Depression	depressiv	Utmattning	utmattad	Skuldkänslor	Sömnlöshet
Switzerland	CH	de (63%)	Angst	ängstlich	Niedergeschlagenheit	niedergeschlagen	Depression	depressiv	Erschöpfung	erschöpft	Schuldgefühle	Schlaflosigkeit
Switzerland	CH	fr (23%)	anxiété	anxieux	abattement	abattu	dépression	déprimé	épuisement	épuisé	culpabilité	insomnie
Switzerland	CH	it (8%)	paura	spaventato	abbattimento	giù	depressione	depressivo	esaurimento	esaurito	senso di colpa	insonnia
United Kingdom	GB	gb	anxiety	anxious	dejection	depressed	depression	depressed	exhaustion	exhausted	guilt	insomnia

Country	listlessness	listlessness	listless	loss of appetite	loss of appetite	loss of appetite	loss of libido	depressed mood	sad mood	bad mood	panic attack	sadness
Austria	Antriebslosigkeit	Unlust	antriebslos	Appetitlosigkeit	Appetitverlust	appetitlos	Libidoverlust	gedrückte Stimmung	traurige Stimmung	schlechte Stimmung	Panikattacke	Traurigkeit
Belgium	Listeloosheid	Onwilligheid	lusteloos	Verlies van eetlust	Verlies van eetlust	appetijtelijk	Libidoverlust	gedeprimeerde stemming	droevige stemming	slecht humeur	Paniek aanval	Droefheid
Belgium	apathie	apathie	apathique	perte d'appétit	perte d'appétit	perte d'appétit	perte de libido	l'humeur dépressive	l'humeur triste	mauvaise humeur	crise de panique	tristesse
Belgium	Antriebslosigkeit	Unlust	antriebslos	Appetitlosigkeit	Appetitverlust	appetitlos	Libidoverlust	gedrückte Stimmung	traurige Stimmung	schlechte Stimmung	Panikattacke	Traurigkeit
Bulgaria	апатия	апатия	безразличен	загуба на апетит	загуба на апетит	загуба на апетит	загуба на либидо	depressed mood	тъжно настроение	лошо настроение	паническа атака	тъга
Croatia	tromost	raditi nevoljko	bez pogona	nedostatak apetita	Gubitak apetita	nedostatak apetita	Gubitak libida	tmurno raspoloženje	tužno raspoloženje	Loše raspoloženje	Napad panike	žalost
Cyprus	άτονη	δυσθυ ία	άτονη	Απώλεια της όρεξης	Απώλεια της όρεξης	χωρίς όρεξη	Απώλεια λί πιντο	καταθλιπτική διάθεση	θλιβερή διάθεση	κακή διάθεση	Επίθεση πανικού	Θλίψη
Cyprus	güçsüz	isteksiz	güçsüz	iştahsızlık	İştah kaybı	iştah kaybı	Libido kaybı	kasvetli ruh hali	üzgün ruh hali	moral bozukluğu	panik atak	üzüntü
Czechia	apatický	Neochota	apatický	nechutenství	Ztráta chuti k jídlu	bez chuti k jídlu	Ztráta libida	depresivní nálada	smutná nálada	špatná nálada	Záchvat paniky	Smutek
Denmark	Løsrivelse	Uvilje	sløv	Tab af appetit	Tab af appetit	uden appetit	Libido tab	deprimeret stemning	trist stemning	dårligt stemning	Panikanfald	Tristhed
Estonia	Lootusetus	Soovimatus	lootusetu	Söögiisu kadumine	Söögiisu kadumine	ilma isuta	Libiido kadumine	depressiivne meeleolu	kurb meeleolu	halb tuju	Paanikahoog	Kurbus
Finland	Luistamattomuus	Haluttomuus	haluttomia	Ruokahaluttomuus	Ruokahaluttomuus	ilman ruokahalua	Libidon menetys	masentunut mieliala	surullinen mieliala	huono mieliala	Paniikkikohtaus	Suru
Finland	Löst tillstånd	Ovilja	listlös	Aptitlöshet	Förlust av aptit	utan aptit	Förlust av libido	deprimerat humör	sorgligt humör	dåligt humör	Panikattack	Sorg
France	apathie	apathie	apathique	perte d'appétit	perte d'appétit	perte d'appétit	perte de libido	l'humeur dépressive	l'humeur triste	mauvaise humeur	crise de panique	tristesse
Germany	Antriebslosigkeit	Unlust	antriebslos	Appetitlosigkeit	Appetitverlust	appetitlos	Libidoverlust	gedrückte Stimmung	traurige Stimmung	schlechte Stimmung	Panikattacke	Traurigkeit
Greece	άτονη	δυσθυ ία	άτονη	Απώλεια της όρεξης	Απώλεια της όρεξης	χωρίς όρεξη	Απώλεια λί πιντο	καταθλιπτική διάθεση	θλιβερή διάθεση	κακή διάθεση	Επίθεση πανικού	Θλίψη
Hungary	Járatlanság	Nem hajlandóság	kedvetlen	Étvágytalanság	Az étvágy elvesztése	étvágytalan	Libidóvesztés	depressziós hangulat	szomorú hangulat	rossz hangulat	Pánikroham	Szomorúság
Iceland	Listleysi	Óánægju	óvélknúnir	Lystarleysi	Lystarleysi	án matarlyst	Missir kynhvöt	drungaleg stemning	sorglegt skap	slæmt skap	Kvíðakast	sorg
Ireland	listlessness	listlessness	listless	loss of appetite	loss of appetite	loss of appetite	loss of libido	depressed mood	sad mood	bad mood	panic attack	sadness

Country	listlessness	listlessness	listless	loss of appetite	loss of appetite	loss of appetite	loss of libido	depressed mood	sad mood	bad mood	panic attack	sadness
Ireland	Gan liosta	Míshásamh	neamh-chumhachta	Cailliúint goile	Cailliúint goile	gan goile	Cailliúint libido	giúmar gruama	giúmar brónach	droch-ghiúmar	Ionsaí scaoll	brón
Italy	svogliatezza	indisponibilità	svogliato	perdita di appetito	perdita di appetito	senza appetito	perdita della libido	umore depresso	umore triste	cattivo umore	attacco di panico	tristezza
Latvia	Bezdarbība	Nevēlēšanās	antriebslos	Apetīta zudums	Apetīta zudums	appetitlos	Libido zudums	gedrückte Stimmung	skumjš noskaņojums	slikts garastāvoklis	Panikas lēkme	Skumjas
Liechtenstein	Antriebslosigkeit	Unlust	antriebslos	Appetitlosigkeit	Appetitverlust	appetitlos	Libidoverlust	gedrückte Stimmung	traurige Stimmung	schlechte Stimmung	Panikattacke	Traurigkeit
Lithuania	Beviltiškumas	Nenoras	beviltiškas	Apetito praradimas	Apetito praradimas	be apetito	Libido praradimas	prislėgta nuotaika	liūdna nuotaika	bloga nuotaika	Panikos priepuolis	Liūdesys
Luxembourg	apathie	apathie	apathique	perte d'appétit	perte d'appétit	perte d'appétit	perte de libido	l'humeur dépressive	l'humeur triste	mauvaise humeur	crise de panique	tristesse
Luxembourg	Listlessness	Onzefriddenheet	onmuecht	Verloscht un Appetit	Verloscht un Appetit	ouni Appetit	Verloscht vun der Libido	düster Stëmmung	traureg Stëmmung	schlecht Stëmmung	Panikattack	Trauregkeet
Luxembourg	Antriebslosigkeit	Unlust	antriebslos	Appetitlosigkeit	Appetitverlust	appetitlos	Libidoverlust	gedrückte Stimmung	traurige Stimmung	schlechte Stimmung	Panikattacke	Traurigkeit
Malta	Nieqes mill-listi	Disgust	bla saħħa	Telf ta 'aptit	Telf ta 'aptit	mingħajr aptit	Telf tal-libido	burdata skura	burdata diqa	burdata ħażina	Attakk ta 'paniku	dwejjaq
Malta	listlessness	listlessness	listless	loss of appetite	loss of appetite	loss of appetite	loss of libido	depressed mood	sad mood	bad mood	panic attack	sadness
Netherlands	Listeloosheid	Onwilligheid	lusteloos	Verlies van eetlust	Verlies van eetlust	appetijtelijk	Libidoverlust	gedeprimeerde stemming	droevige stemming	slecht humeur	Paniek aanval	Droefheid
Norway	sløvhet	motvilje	sløv	tap av Appetit	tap av Appetit	uten appetitt	tap av lobido	deprimert humør	trist humør	dårlig humør	panikkanfall	tristhet
Poland	Bezczynność	Niechęć	bezsenny	Utrata apetytu	Utrata apetytu	bez apetytu	Utrata libido	przygnębiony nastrój	smutny nastrój	z y nastrój	Atak paniki	Smutek
Portugal	Apatia	Relutância	sem energia	Perda de apetite	Perda de apetite	sem apetite	Perda de libido	humor sombrio	humor triste	mau humor	Ataque de pânico	tristeza
Romania	Apatie	Reticenta	neputernic	Pierderea poftei de mâncare	Pierderea poftei de mâncare	fără apetit	Pierderea libidoului	dispoziție mohorâtă	trist	stare rea de spirit	Atac de panică	tristeţe
Slovakia	Bezočivosť	Neochota	bez sily	Strata chuti do jedla	Strata chuti do jedla	bez chuti do jedla	Strata libida	pochmúrna nálada	smutná nálada	zlá nálada	Záchvat paniky	smútok
Slovenia	brezvoljnost	nenaklonjenost	brez moči	izguba apetita	izguba apetita	brez apetita	izguba libida	mračno razpoloženje	žalostno razpoloženje	slaba volja	panični napad	žalost
Spain	languidez	reluctancia	sin poder	pérdida de apetito	pérdida de apetito	sin apetito	pérdida de libido	estado de ánimo sombrío	estado de ánimo triste	mal humor	ataque de pánico	tristeza
Sweden	Löst tillstånd	Ovilja	listlös	Aptitlöshet	Förlust av aptit	utan aptit	Förlust av libido	deprimerat humör	sorgligt humör	dåligt humör	Panikattack	Sorg
Switzerland	Antriebslosigkeit	Unlust	antriebslos	Appetitlosigkeit	Appetitverlust	appetitlos	Libidoverlust	gedrückte Stimmung	traurige Stimmung	schlechte Stimmung	Panikattacke	Traurigkeit
Switzerland	apathie	apathie	apathique	perte d'appétit	perte d'appétit	perte d'appétit	perte de libido	l'humeur dépressive	l'humeur triste	mauvaise humeur	crise de panique	tristesse
Switzerland	svogliatezza	indisponibilità	svogliato	perdita di appetito	perdita di appetito	senza appetito	perdita della libido	umore depresso	umore triste	cattivo umore	attacco di panico	tristezza
United Kingdom	listlessness	listlessness	listless	loss of appetite	loss of appetite	loss of appetite	loss of libido	depressed mood	sad mood	bad mood	panic attack	sadness

Country	sad	sleep disorder	sleep problem	sleepless	weariness	tired	worthlessness	worthless	psychiatry	psychology	psychotherapy	psychological treatment
Austria	traurig	Schlafstörung	Schlafproblem	schlaflos	Müdigkeit	müde	Wertlosigkeit	wertlos	Psychiatrie	Psychologie	Psychotherapie	Psychologische Behandlung
Belgium	sad	Slaapstoornis	Slaapprobleem	slapeloos	Vermoeidheid	moe	Waardeloosheid	waardeloze	Psychiatrie	Psychologie	Psychotherapie	psychologische behandeling
Belgium	triste	troubles du sommeil	problèmes de sommeil	insomnie	fatigue	fatigué	inutilité	sans valeur	psychiatrie	psychologie	psychothérapie	traitement psychologique
Belgium	traurig	Schlafstörung	Schlafproblem	schlaflos	Müdigkeit	müde	Wertlosigkeit	wertlos	Psychiatrie	Psychologie	Psychotherapie	Psychologische Behandlung
Bulgaria	тъжен	sleep disorder	проблем със съня	безсънна	умора	уморен	безполезност	безполезен	психиатър	психология	психотерапия	психологическо лечение
Croatia	žalostan	Poremećaj spavanja	Problem sa spavanjem	nesanica	umor	umoran	bezvrijednost	bezvrijedan	psihijatrija	psihologija	psihoterapija	Psihološki tretman
Cyprus	θλιβερό	Διαταραχή ύπνου	Πρόβλη α ύπνου	άγρυπνος	Κόπωση	κουρασ ένος	Αχρηστία	wertlos	Ψυχιατρική	Ψυχολογία	Ψυχοθεραπεία	Ψυχολογική θεραπεία
Cyprus	üzgün	uyuma bozuklukları	uyku sorunu	uykusuz	yorgunluk	uykulu	değersizlik	değersiz	psikiyatri	psikoloji	psikoterapi	psikolojik tedavi
Czechia	smutný	Porucha spánku	Problém se spánkem	bezesný	Únava	unavený	Bezcennost	bezcenné	Psychiatrie	psychologie	psychoterapie	Psychologická léčba
Denmark	trist	Schlafstörung	Søvnproblem	schlaflos	Træthed	træt	Værdiløshed	værdiløs	Psykiatri	Psykologi	Psykoterapi	Psykologisk behandling
Estonia	kurb	Unehäire	Uneprobleem	unetu	Väsimus	väsinud	Väärtusetus	väärtusetu	Psühhiaatria	Psühholoogia	Psühhoteraapia	Psühholoogiline ravi
Finland	surullinen	Unihäiriö	Nukkumisongelma	uneton	Väsymys	väsynyt	Arvottomuus	arvoton	Psykiatria	Psykologia	Psykoterapia	Psykologinen hoito
Finland	ledsen	Sömnstörning	Sömnproblem	sömnlös	Trötthet	trött	Värdelöshet	värdelösa	Psykiatri	Psykologi	Psykoterapi	Psykologisk behandling
France	triste	troubles du sommeil	problèmes de sommeil	insomnie	fatigue	fatigué	inutilité	sans valeur	psychiatrie	psychologie	psychothérapie	traitement psychologique
Germany	traurig	Schlafstörung	Schlafproblem	schlaflos	Müdigkeit	müde	Wertlosigkeit	wertlos	Psychiatrie	Psychologie	Psychotherapie	Psychologische Behandlung
Greece	θλιβερό	Διαταραχή ύπνου	Πρόβλη α ύπνου	άγρυπνος	Κόπωση	κουρασ ένος	Αχρηστία	wertlos	Ψυχιατρική	Ψυχολογία	Ψυχοθεραπεία	Ψυχολογική θεραπεία
Hungary	traurig	Alvászavar	Alvási probléma	álmatlan	Fáradtság	fáradt	Értéktelenség	értéktelen	Pszichiátria	Pszichológia	Pszichoterápia	Pszichológiai kezelés
Iceland	Dapur	Svefnröskun	Svefnvandamál	svefnlaus	þreyta	þreyttur	einskis virði	verðlaus	geðlækningar	sálfræði	sálfræðimeðferð	Sálfræðileg meðferð
Ireland	sad	sleep disorder	sleep problem	sleepless	weariness	tired	worthlessness	worthless	psychiatry	psychology	psychotherapy	psychological treatment

Country	sad	sleep disorder	sleep problem	sleepless	weariness	tired	worthlessness	worthless	psychiatry	psychology	psychotherapy	psychological treatment
Ireland	Brón	Neamhord codlata	Neamhord codlata	gan codladh	tuirse	tuirseach	fiúntas	gan fiúntas	síciatracht	síceolaíocht	síciteiripe	Cóireáil síceolaíoch
Italy	triste	disturbo del sonno	problema di sonno	insonne	fatica	stanco	inutilità	inutile	psichiatria	psicologia	psicoterapia	trattamento psicologico
Latvia	skumji	Miega traucējumi	Miega problēma	bezmiegs	Nogurums	müde	Bezvērtība	bezvērtīgs	Psihiatrija	Psiholoģija	Psihoterapija	Psiholoģiskā ārstēšana
Liechtenstein	traurig	Schlafstörung	Schlafproblem	schlaflos	Müdigkeit	müde	Wertlosigkeit	wertlos	Psychiatrie	Psychologie	Psychotherapie	Psychologische Behandlung
Lithuania	liūdna	Miego sutrikimas	Miego problema	be miego	Nuovargis	pavargęs	Bevertiškumas	bevertis	Psichiatrija	Psichologija	Psichoterapija	Psichologinis gydymas
Luxembourg	triste	troubles du sommeil	problèmes de sommeil	insomnie	fatigue	fatigué	inutilité	sans valeur	psychiatrie	psychologie	psychothérapie	traitement psychologique
Luxembourg	Traureg	Schlofstéierungen	Schlofproblem	schloflos	Middegkeet	midd	wäertlosegkeet	wäertlos	Psychiatrie	Psychologie	Psychotherapie	Psychologesch Behandlung
Luxembourg	traurig	Schlafstörung	Schlafproblem	schlaflos	Müdigkeit	müde	Wertlosigkeit	wertlos	Psychiatrie	Psychologie	Psychotherapie	Psychologische Behandlung
Malta	Imdejjaq	Disturbi fl-irqad	Problema ta 'rqad	bla rqad	għeja	għajjien	inutilità	siwi	psikjatrija	psikoloġija	psikoterapija	Trattament psikoloġiku
Malta	sad	sleep disorder	sleep problem	sleepless	weariness	tired	worthlessness	worthless	psychiatry	psychology	psychotherapy	psychological treatment
Netherlands	sad	Slaapstoornis	Slaapprobleem	slapeloos	Vermoeidheid	moe	Waardeloosheid	waardeloze	Psychiatrie	Psychologie	Psychotherapie	psychologische behandeling
Norway	lei seg	søvnforstyrrelse	søvnproblem	søvnløs	utmattelse	trett	verdiløshet	verdiløs	psykiatri	psykologi	psykoterapi	psykologisk behandling
Poland	traurig	Zaburzenia snu	Problem ze snem	bezsenny	Zmęczenie	zmęczony	Bezwartościowość	bezużyteczny	Psychiatria	Psychologia	Psychoterapia	Leczenie psychologiczne
Portugal	Triste	Distúrbio do sono	Problema de dormir	sem dormir	fadiga	cansado	inutilidade	inútil	psiquiatria	psicologia	psicoterapia	Tratamento psicológico
Romania	Trist	Probleme cu somnul	Problema somnului	nedormită	oboseală	obosit	lipsa de valoare	fără valoare	psihiatrie	psihologie	psihoterapie	Tratamentul psihologic
Slovakia	Smutné	Porucha spánku	Problém so spánkom	Nevyspatý	únava	unavený	bezcennosť	bezcenný	psychiatria	psychológia	psychoterapia	psychologická liečba
Slovenia	Žalostno	motnje spanja	težava s spanjem	neprespan	utrujenost	utrujen	ničvrednost	brez vrednosti	psihiatrija	psihologije	psihoterapija	psihološko zdravljenje
Spain	triste	trastorno del sueño	problema de sueño	insomnio	fatiga	cansado	inutilidad	sin valor	psiquiatría	psicología	psicoterapia	tratamiento psicológico
Sweden	ledsen	Sömnstörning	Sömnproblem	sömnlös	Trötthet	trött	Värdelöshet	värdelösa	Psykiatri	Psykologi	Psykoterapi	Psykologisk behandling
Switzerland	traurig	Schlafstörung	Schlafproblem	schlaflos	Müdigkeit	müde	Wertlosigkeit	wertlos	Psychiatrie	Psychologie	Psychotherapie	Psychologische Behandlung
Switzerland	triste	troubles du sommeil	problèmes de sommeil	insomnie	fatigue	fatigué	inutilité	sans valeur	psychiatrie	psychologie	psychothérapie	traitement psychologique
Switzerland	triste	disturbo del sonno	problema di sonno	insonne	fatica	stanco	inutilità	inutile	psichiatria	psicologia	psicoterapia	trattamento psicologico
United Kingdom	sad	sleep disorder	sleep problem	sleepless	weariness	tired	worthlessness	worthless	psychiatry	psychology	psychotherapy	psychological treatment

Country	psychologist	psychologist	psychotherapist	psychotherapist
Austria	Psychologe	Psychologin	Psychotherapeut	Psychotherapeutin
Belgium	Psycholoog	Psycholoog	Psychotherapeut	Psychotherapeut
Belgium	psychologue	psychologue	psychothérapeute	psychothérapeute
Belgium	Psychologe	Psychologin	Psychotherapeut	Psychotherapeutin
Bulgaria	психолог	психолог	психотерапевт	психотерапевт
Croatia	psiholog	psihološkinja	Psychotherapeut	psihoterapeutkinja
Cyprus	Ψυχολόγος	Ψυχολόγος	Ψυχοθεραπευτής	Ψυχοθεραπευτής
Cyprus	psikolog	psikolog	psikoterapist	psikoterapist
Czechia	psycholog	psycholog	psychoterapeut	Psychoterapeut
Denmark	Psykolog	Psykolog	Psykoterapeut	Psykoterapeut
Estonia	Psühholoog	Psühholoog	Psühhoterapeut	Psühhoterapeut
Finland	Psykologi	Psykologi	Psykoterapeutti	Psykoterapeutti
Finland	Psykolog	Psykolog	Psykoterapeut	Psykoterapeut
France	psychologue	psychologue	psychothérapeute	psychothérapeute
Germany	Psychologe	Psychologin	Psychotherapeut	Psychotherapeutin
Greece	Ψυχολόγος	Ψυχολόγος	Ψυχοθεραπευτής	Ψυχοθεραπευτής
Hungary	Psychologe	Psychologin	Pszichoterapeuta	Pszichoterapeuta
Iceland	sálfræðingur	sálfræðingur	sálfræðingur	Sálfræðingur
Ireland	psychologist	psychologist	psychotherapist	psychotherapist

Country	psychologist	psychologist	psychotherapist	psychotherapist
Ireland	síceolaí	síceolaí	síciteiripeoir	Síciteiripeoir
Italy	psicologo	psicologa	psicoterapeuta	psicoterapeuta
Latvia	Psihologs	Psihologs	Psychotherapeut	Psihoterapeits
Liechtenstein	Psychologe	Psychologin	Psychotherapeut	Psychotherapeutin
Lithuania	Psichologas	Psichologas	Psichoterapeutas	Psichoterapeutas
Luxembourg	psychologue	psychologue	psychothérapeute	psychothérapeute
Luxembourg	Psycholog	Psycholog	Psychotherapeut	Psychotherapeut
Luxembourg	Psychologe	Psychologin	Psychotherapeut	Psychotherapeutin
Malta	psikologu	psikologu	psikoterapista	Psikoterapista
Malta	psychologist	psychologist	psychotherapist	psychotherapist
Netherlands	Psycholoog	Psycholoog	Psychotherapeut	Psychotherapeut
Norway	psykolog	psykolog	Psykoterapeut	Psykoterapeut
Poland	Psychologe	Psycholog	Psychoterapeuta	Psychoterapeuta
Portugal	psicólogo	psicólogo	psicoterapeuta	Psicoterapeuta
Romania	psiholog	psiholog	psihoterapeut	Psihoterapeut
Slovakia	psychológ	psychológ	psychoterapeut	Psychoterapeut
Slovenia	psihologinja	psihologinja	psihoterapevt	Psihoterapevt
Spain	psicólogo	psicóloga	psicoterapeuta	psicoterapeuta
Sweden	Psykolog	Psykolog	Psykoterapeut	Psykoterapeut
Switzerland	Psychologe	Psychologin	Psychotherapeut	Psychotherapeutin
Switzerland	psychologue	psychologue	psychothérapeute	psychothérapeute
Switzerland	psicologo	psicologa	psicoterapeuta	psicoterapeuta
United Kingdom	psychologist	psychologist	psychotherapist	psychotherapist
