# Supplementary material for: Impact of the pandemic and its containment measures in Europe upon aspects of affective impairments: a Google Trends informetrics study
Source: Psychol Med. 2023 Jun 26;53(16):7685–97. doi: 10.1017/S0033291723001563 (PMC10755220; doi:10.1017/S0033291723001563)
Supplement: Szilagyi et al. supplementary material 3 — Szilagyi et al. supplementary material [file S0033291723001563sup003.pdf]

Impact of the Pandemic and its Containment Measures in Europe upon Aspects of Affective Impairments: A Google Trends Informetrics Study

```
#
# google trends data acquisition
#
# version 0.1 - nov. 2022
#

import pandas
import time
import sys
from pytrends.request import TrendReq
from datetime import date


#
# the query time frames
#
timeframe = [
   '2016-07-01 2019-06-30', # 1/2 time frame
   '2019-07-01 2022-06-30', # 2/2 time frame
   '2016-07-01 2017-06-30', # 1. time frame Q3-16, Q4-16, Q1-17, Q2-17,
   '2017-07-01 2018-06-30', # 2. time frame Q3-17, Q4-17, Q1-18, Q2-18,
   '2018-07-01 2019-06-30', # 3. time frame Q3-18, Q4-18, Q1-19, Q2-19,
   '2019-07-01 2020-06-30', # 4. time frame Q3-19, Q4-19, Q1-20, Q2-20,
   '2020-07-01 2021-06-30', # 5. time frame Q3-20, Q4-20, Q1-21, Q2-21,
   '2021-07-01 2022-06-30'  # 6. time frame Q3-21, Q4-21, Q1-22, Q2-22,
]

#
# the query terms (english version)
#
keywords = [
   'anxiety',                            'anxious',                   'dejection',
   'depressed',                          'depression',                'depressed',
   'exhaustion',                         'exhausted',                 'guilt',
   'insomnia',                           'listlessness',              'listlessness (alternative)',
   'listless',                           'loss of appetite',          'loss of appetite (alternative 1)',
   'loss of appetite (alternative 2)',   'loss of libido',            'depressed mood',
   'sad mood',                           'bad mood',                  'panic attack',
   'sadness',                            'sad',                       'sleep disorder',
   'sleep problem',                      'sleepless',                 'weariness',
   'tired',                              'worthlessness',             'worthless',
   'psychiatry',                         'psychology',                'psychotherapy',
   'psychological treatment',            'psychologist (male)',       'psychologist (female)',
   'psychotherapist (male)',             'psychotherapist (female)'
]

#
# the query terms (the non-English translations are stored in a CSV file):
#
# file content:
# "Country",  "Country-Code","Language", "Usage", "anxiety",    "anxious",   "dejection",             ...
# "Austria",  "AT",           "DE",       1.00,    "Angst",      "ängstlich", "Niedergeschlagenheit", ...
# "Belgium",  "BE",           "NL",       0.59,    "Angst",      "bang",      "Neerslachtigheid",     ...
# "Belgium",  "BE",           "FR",       0.40,    "anxiété",    "anxieux",   "abattement",           ...
# "Belgium",  "BE",           "DE",       0.01,    "Angst",      "ängstlich", "Niedergeschlagenheit", ...
# "Bulgaria", ...
# ...
#
# see Appendix B for details on query terms and translations
#
queryTerms = pandas.read_csv('./Search_Terms.csv')
print(queryTerms)

#
# script execution date and time
#
startTime = time.time()
execDay = date.today().strftime("%Y-%m-%d")
print(execDay)
```

by Istvan-Szilard Szilagyi, Eva Eggeling, Helmar Bornemann-Cimenti, Torsten Ullrich

```
#
# the main functionalty is implemented in the 'googleTrends' routine:
# parameter 'output'       : a filename; the corresponding file will be created
#                            and the query results will be written into this file.
# parameter 'querytime'    : the time range to query
#
# parameter 'querydetails' : one row of the queryTerms (CSV file)
#
def googleTrends(output, querytime, querydetails):
   print('   generating ' + output)
   #
   # create query request
   #
   pytrend = TrendReq(hl=querydetails['Language'], tz=360, timeout=(10,25))
   #
   # the resulting dataset
   #
   dataset = []
   gotData = False
   #
   # iterate over all query terms (stored in keywords)
   #
   for key in keywords:
      #
      # get query term translation (stored in querydetails)
      #
      print("    {} - {}".format(key, querydetails[key]))
      querykeywords = [ querydetails[key] ]
      googlerequest = 0
      googlesuccess = False
      #
      # try several time to perform query request
      #
      while not(googlesuccess):
         try:
            pytrend.build_payload(kw_list = querykeywords, timeframe = querytime,
                                  geo = querydetails['Country-Code'])
            data = pytrend.interest_over_time()
            googlesuccess = True
         except:
            googlerequest = googlerequest + 1
            if googlerequest > 5:
               raise Exception("Blocked by Google :-(")
            print("    /// retry ///")
            time.sleep(10)
      #
      # add results to dataset or warn about empty answer
      #
      if not data.empty:
         dataset.append(data)
         gotData = True
      else:
         print('ERROR - no data for "{}" in {}'.format(key, querydetails['Country-Code']))
   #
   # store results
   #
   if gotData:
      result = pandas.concat(dataset, axis=1)
      result.to_csv(output)
   print('   finished ' + output)
   return

#
# main function:
#
processingStep  = 0;
#
# iterate over all time ranges and query terms:
#
for timeindex, querytime in enumerate(timeframe):
   for index, contents in queryTerms.iterrows():

      print("processing step {}".format(processingStep))
      print("    {} - {}".format(contents['Country-Code'], contents['Language']))

      processingStep = processingStep + 1;
      outputfile = ('result_' + contents['Country-Code'] + '_' + contents['Language'] + '_'
                    + str(timeindex) + '_' + str(processingStep - 1) + '_' + execDay  + '.csv')

      googleTrends(outputfile, querytime, contents)
```
